# Supplementary material for: Decreasing exciton dissociation rates for reduced voltage losses in organic solar cells
Source: Nat Commun. 2024 Mar 27;15:2693. doi: 10.1038/s41467-024-46797-5 (PMC10973424; doi:10.1038/s41467-024-46797-5)
Supplement: Supplementary file 1 — Supplementary Information [file 41467_2024_46797_MOESM1_ESM.pdf]

Supplementary Information

Decreasing Exciton Dissociation Rates for Reduced Voltage Losses in Organic Solar Cells

Hongbo Wu[1], Hao Lu[2], Yungui Li[3], Xin Zhou[3], Guanqing Zhou[4], Hailin Pan[1], Hanyu Wu[1], Xunda Feng[1], Feng Liu[4], Koen Vandewal[5], Wolfgang Tress[6], Zaifei Ma[1,*], Zhishan Bo[2,*], and Zheng Tang[1,*]

1. State Key Laboratory for Modification of Chemical Fibers and Polymer Materials, Center for Advanced Low-dimension Materials, College of Materials Science and Engineering, Donghua University, Shanghai, 201620, P. R. China
2. Key Laboratory of Energy Conversion and Storage Materials, College of Chemistry, Beijing Normal University, 100875, Beijing, P. R. China
3. Max Planck Institute for Polymer Research, Ackermannweg 10, 55128, Mainz, Germany
4. Frontiers Science Center for Transformative Molecules, School of Chemistry and Chemical Engineering, Shanghai Jiao Tong University, Shanghai 200240, P. R. China
5. Instituut voor Materiaalonderzoek (IMO-IMOMEC), Hasselt University, Wetenschapspark 1, BE-3590 Diepenbeek, Belgium
6. Institute of Computational Physics, Zurich University of Applied Sciences, Wildbachstr. 21, 8401 Winterthur, Switzerland

Emails: mazaifei@dhu.edu.cn; zsbo@bnu.edu.cn; ztang@dhu.edu.cn

Table of contents

Supplementary Figures s2
Supplementary Note 1: Derivation of analytical equations.. s13
Supplementary Note 2: Details about field dependent PL measurements s20
Supplementary Note 2: Details about TPV measurements s22
Supplementary Note 4: Ternary solar cells based on PM7/IT4F/Y5OD s27
Supplementary Note 5: Ternary solar cells based on PM6/fullenere/SM16 .... s30
Supplementary Note 6: Ternary solar cells based on PM7/IT4F/BTA3 .... s33

Supplementary Figures

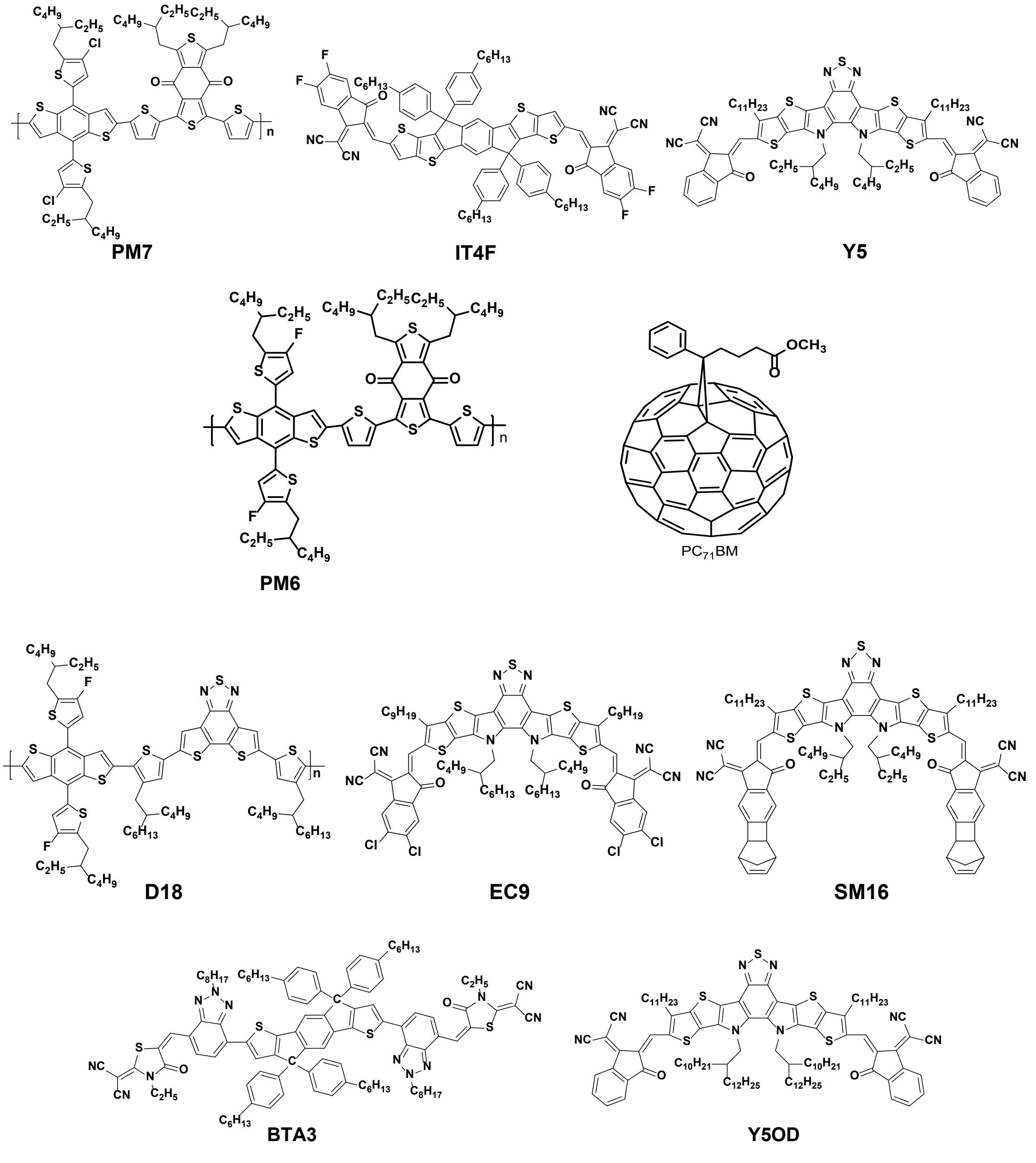

Supplementary Figure 1. Photovoltaic materials. Chemical structures of the donors and acceptors used in this work.

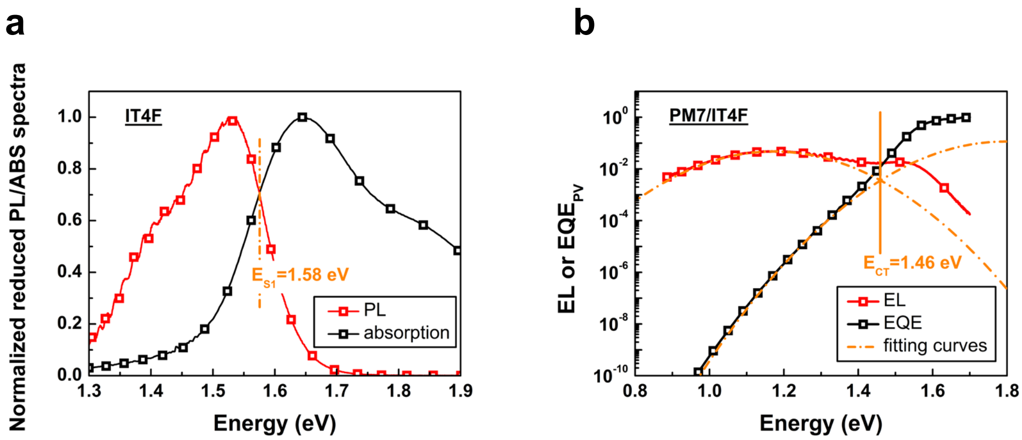

Supplementary Figure 2. Estimation of ΔE_{CT} for the PM7/IT4F binary blend. a) Normalized reduced PL and absorption spectra of IT4F. The energy of the S_1 state (E_{S1}) of IT4F in the blend is determined from the crossing point of the PL and the absorption spectra. **b)** Sensitive EL and EQE_{PV} spectra of the PM7/IT4F solar cell. E_{CT} is determined by a Gaussian fitting to the lower energy part of the EQE_{PV} spectrum using the method described in the literature (Phys. Rev. B 2010, 81, 125204). Accordingly, ΔE_{CT} (= E_{S1} - E_{CT}) of the PM7/IT4F binary blend is determined, which is about 0.12 eV.

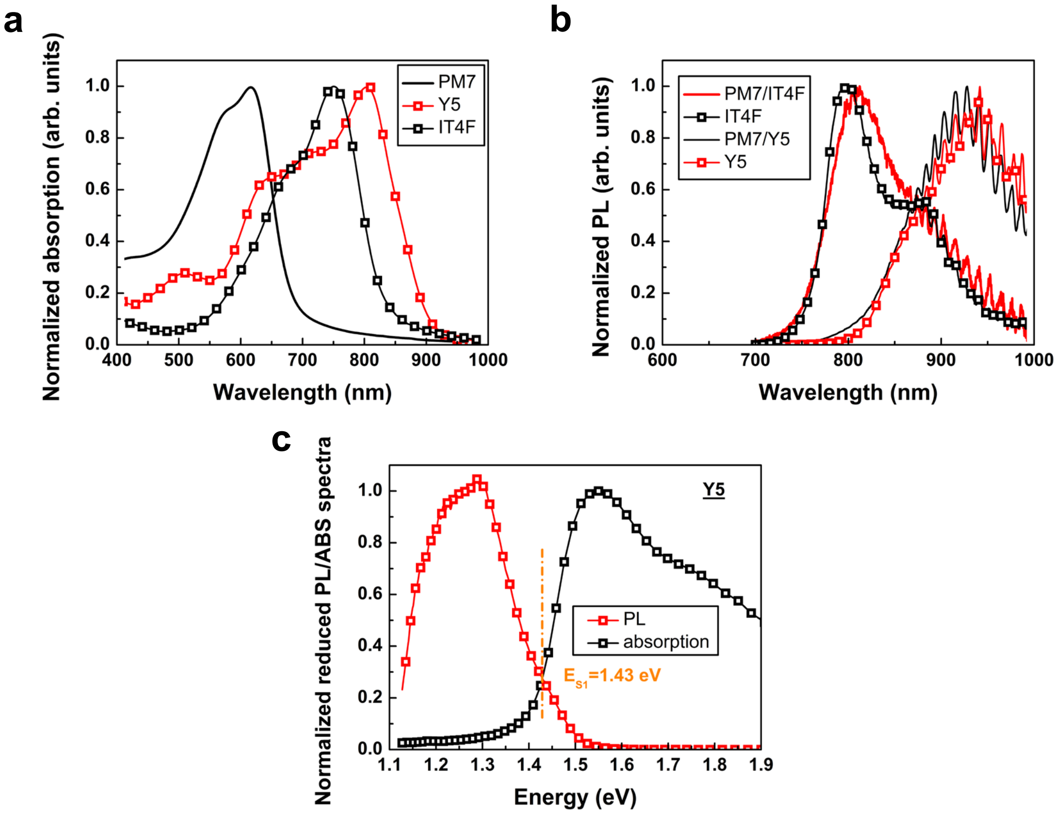

Supplementary Figure 3. Optical properties of PM7, Y5, and IT4F. a) UV-vis absorption spectra of the thin films of IT4F, Y5, and PM7, and **b)** PL spectra of the blend films of PM7/IT4F and PM7/Y5. **c)** Normalized reduced PL and absorption spectra of Y5. The energy of the S_1 state (E_{S1}) of Y5 in the blend is determined from the crossing point of the PL and the absorption spectra.

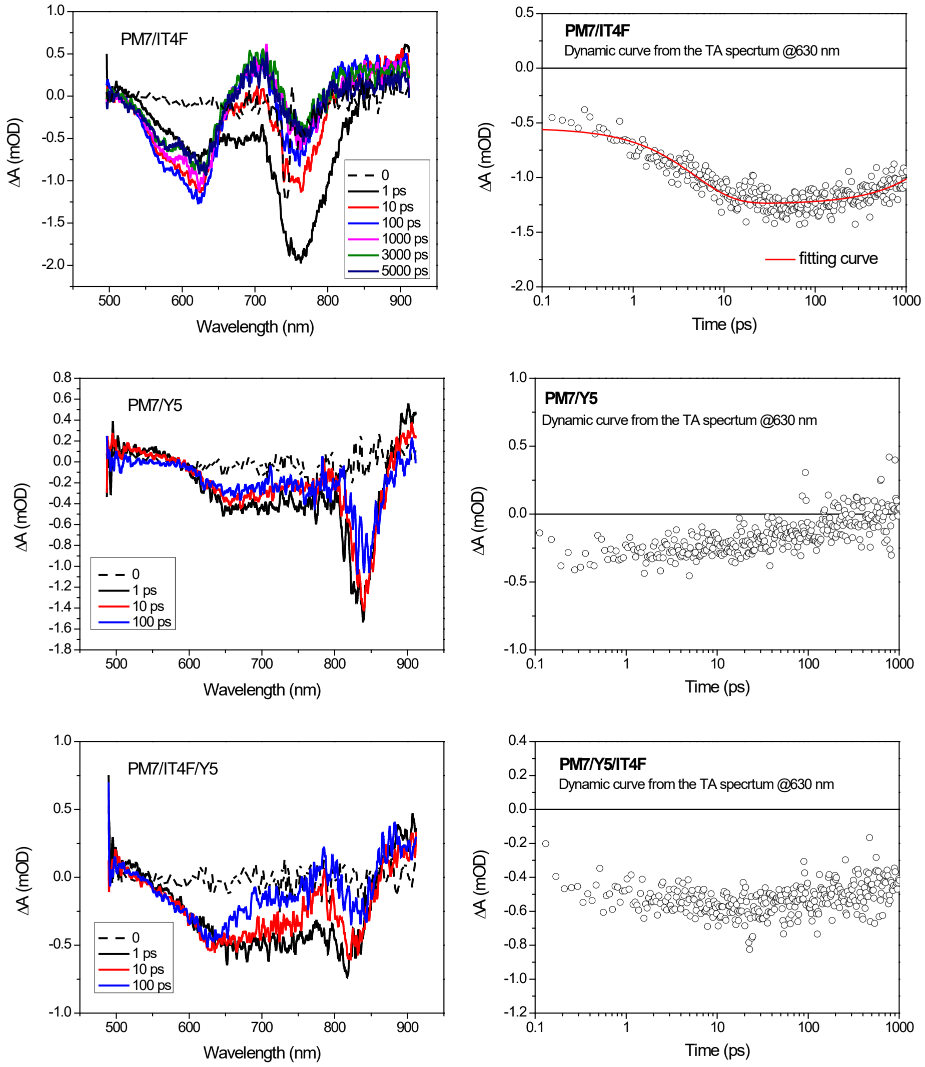

Supplementary Figure 4. Transient absorption spectroscopy on the blend films. TA spectra (left) and time evolution of TA signals recorded at the 630 nm (right), for the blend of PM7/IT4F, PM7/Y5, and PM7/IT4F/Y5 (20%), excited at 750 nm. The fit parameters used for the biexponential fit of the dynamic curve of the PM7/IT4F system include: A_1=-3.65×10^{-4}, t_1=896 ps, A_2=6.35×10^{-4}, and t_2=8.06 ps.

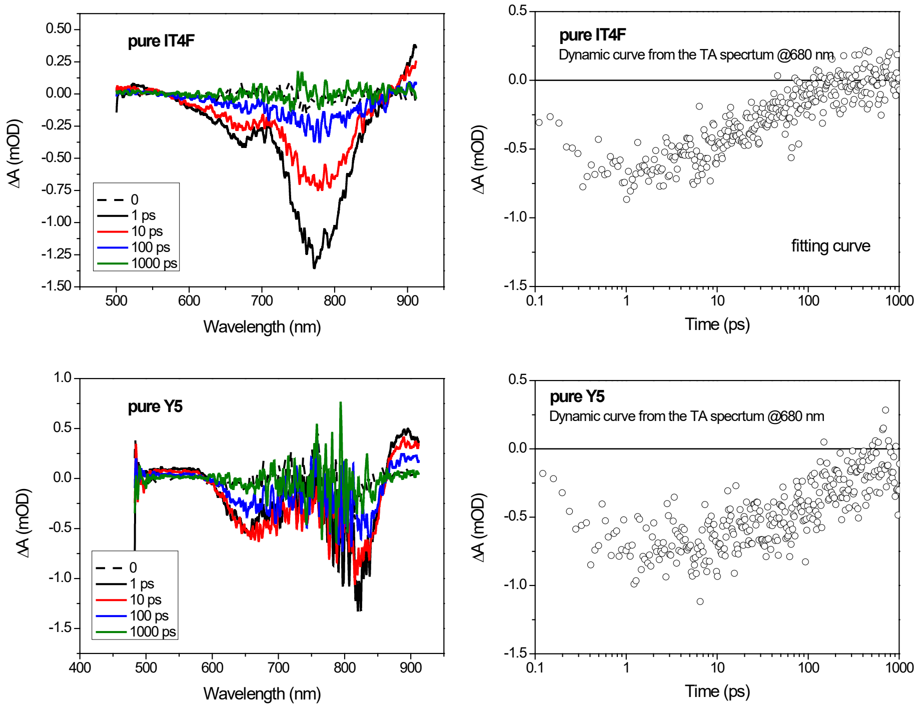

Supplementary Figure 5. Transient absorption spectroscopy on the acceptor films. TA spectra (left) and time evolution of TA signals recorded at the 680 nm (right), for the pure films of IT4F and Y5, excited at 750 nm.

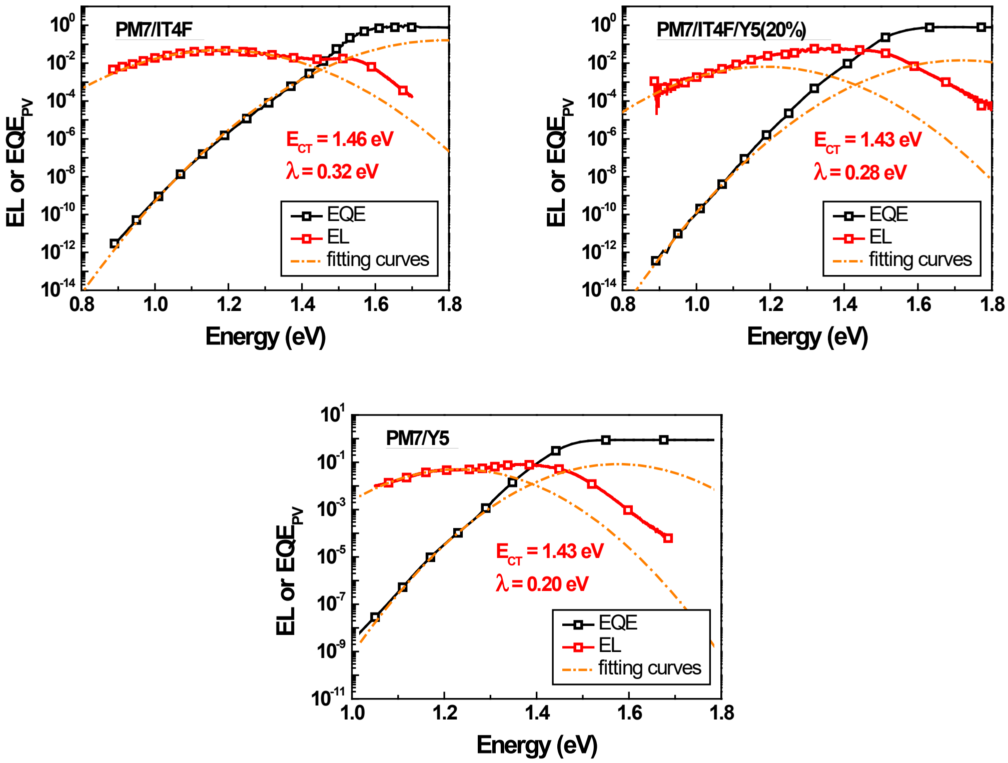

Supplementary Figure 6. Sensitive EL and *EQE_{PV}* spectra of the binary and ternary solar cells. The E_{CT} and reorganization energy (λ) were determined by Gaussian fitting to the lower energy part of the EQE_{PV} spectrum using the method described in the literature (J. Mater. Chem. A, 2021, 9, 19770). The reorganization energy is found to be 0.32 and 0.20 eV for the binary blends of PM7/IT4F and PM7/Y5, respectively, attributed to the different molecular structures of IT4F and Y5, as well as the different morphologies of the PM7/IT4F and PM7/Y5 blends. An increase in Y5 content from 0% to 20% results in a reduction of reorganization energy from 0.32 to 0.28 eV, suggesting that the addition of Y5 could alter the morphology of the active layer.

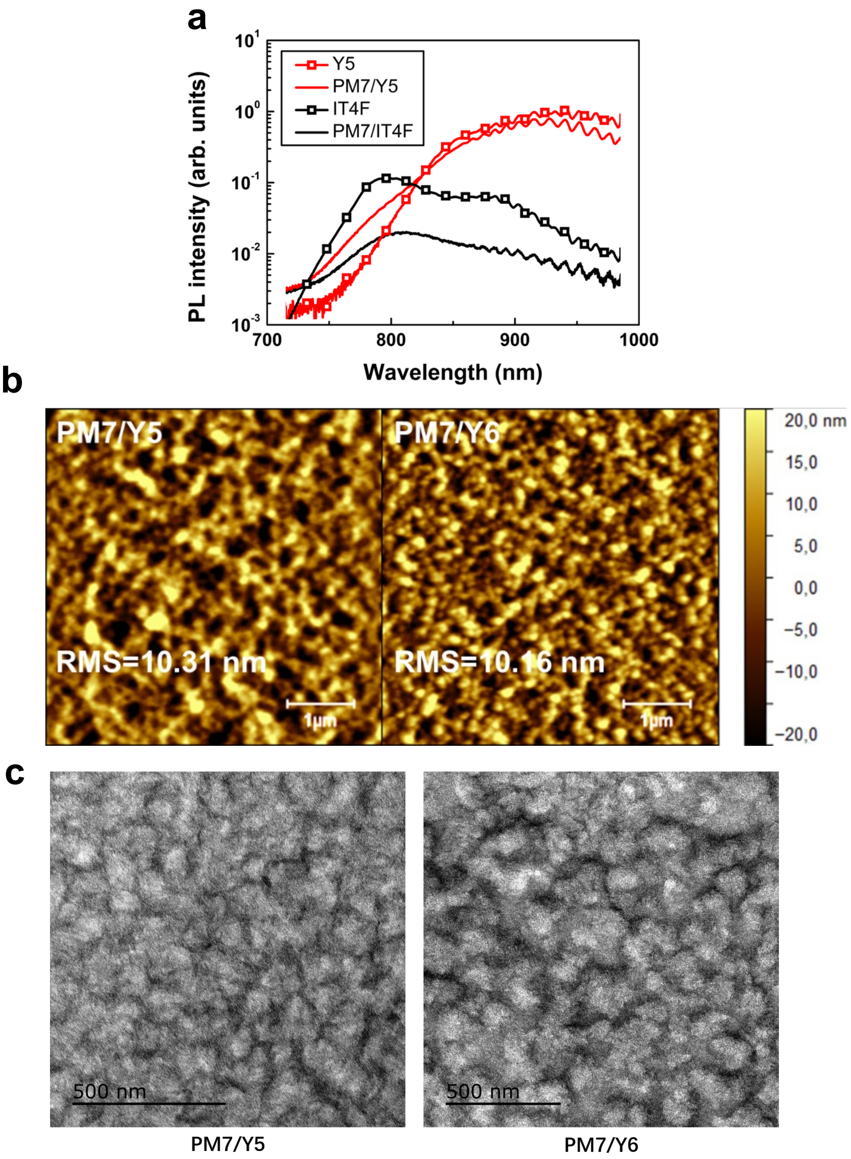

Supplementary Figure 7. Exciton dissociation efficiency in the PM7/Y5 binary blend. a) Steady state PL spectra of the PM7/IT4F and PM7/Y5 binary blends, and the IT4F and Y5 pure films. The PL emission intensity of the PM7/Y5 binary blend film, normalized by the absorbance of the film at the excitation wavelength, is very close to that of the Y5 pure film, implying that the dissociation of the S_1 state of Y5 in the PM7/Y5 blend film is inefficient. **b)** Atomic force microscope (AFM) and **c)** TEM images of the PM7/Y5 and PM7/Y6 binary blends. The AFM and TEM images of the thin films of PM7/Y5 and PM7/Y6 are similar, indicating comparable morphological properties in these blends. Considering the high efficiency of the solar cell based on PM7/Y6, it is anticipated that the morphology of the PM7/Y6 blend is optimized for the diffusion of S_1 states to a donor/acceptor interface. Similarly, the morphology of the PM7/Y5 blend is expected to be optimized. Therefore, the inefficient dissociation of the S_1 state of Y5 in the PM7/Y5 blend is mainly attributed to a low k_{DS}.

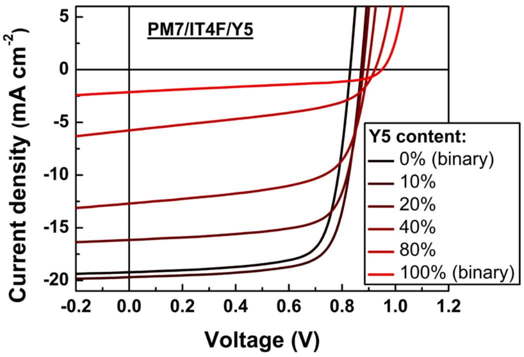

Supplementary Figure 8. Photovoltaic performance of the PM7/IT4F/Y5 ternary solar cells. *J-V* curves are recorded for the Y5 ternary solar cells with different Y5 content.

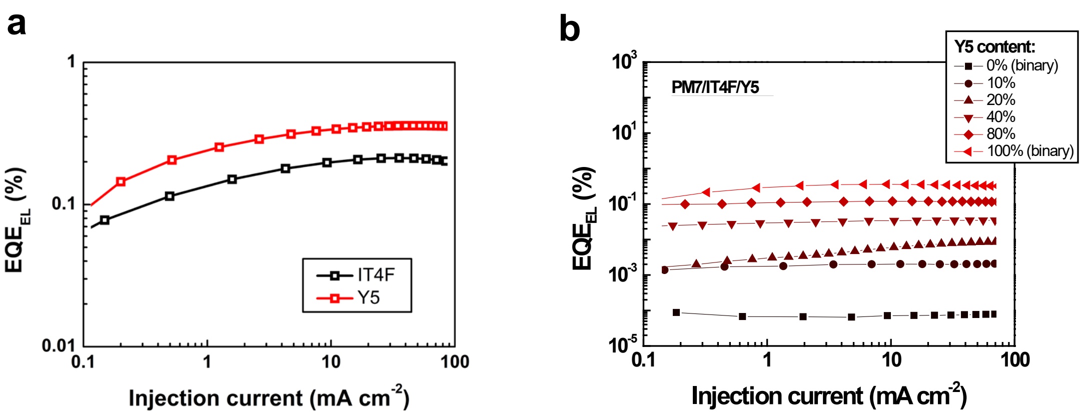

Supplementary Figure 9. Non-radiative voltage losses in ternary solar cells based on IT4F and Y5. **a)** EQE_{EL} of the solar cells based on pure IT4F and pure Y5 plotted as a function of injection current. **b)** EQE_{EL} of the solar cells based on PM7/IT4F/Y5, with different Y5 content, plotted as a function of injection current.

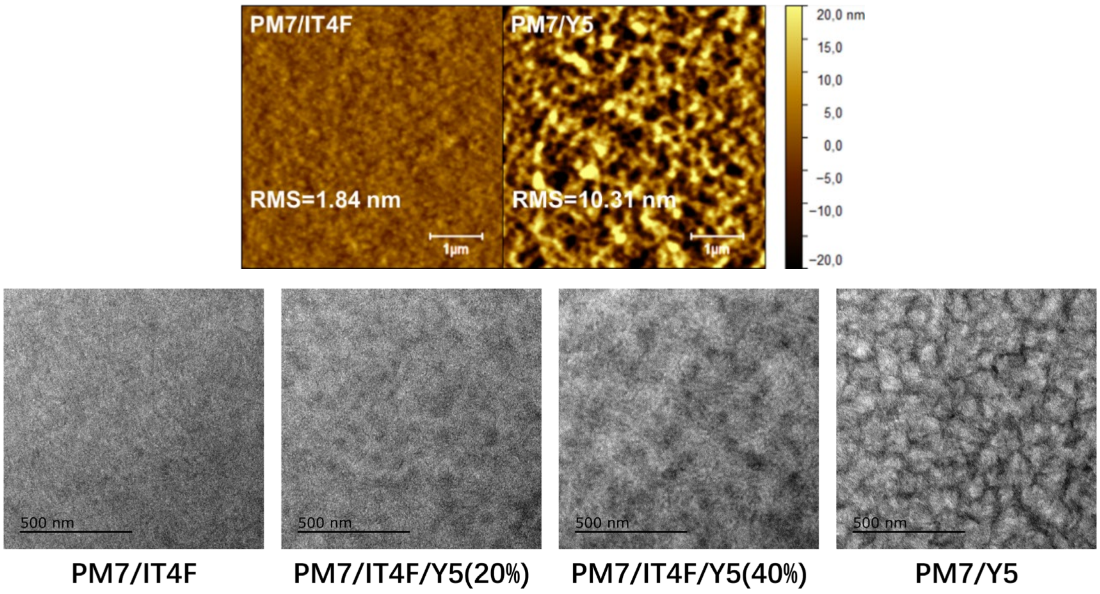

Supplementary Figure 10. AFM and TEM images of the PM7/IT4F and PM7/Y5 blends. The AFM (upper) and TEM (lower) images indicate differences in the morphologies of the active layers based on PM7/IT4F and PM7/Y5. A significant increase in Y5 content in the ternary blend of PM7/IT4F/Y5 results in a morphological change in the active layer.

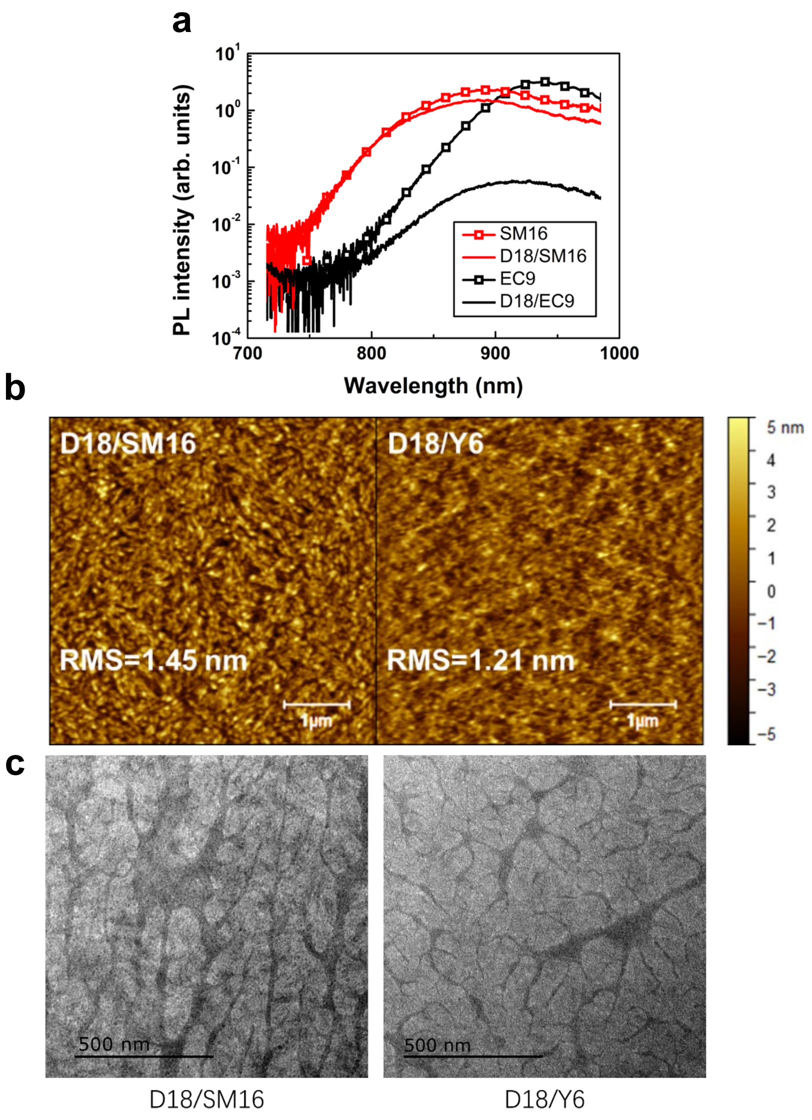

Supplementary Figure 11. Exciton dissociation efficiency in the D18/SM16 binary blend. a) PL spectra of the D18/SM16 and D18/EC9 binary blends, and the SM16 and EC9 pure films. The excitation wavelength used is 500 nm. The PL emission intensity of the D18/SM16 binary blend film, normalized by the absorbance of the film at the excitation wavelength, is very close to that of the SM16 pure film, implying that the dissociation of the S_1 state of SM16 in the D18/SM16 blend film is inefficient. **b)** AFM and **c)** TEM images of the D18/SM16 and D18/Y6 binary blends. From the AFM and TEM images, we find that the morphological characteristics of the D18/SM16 blend are similar to those of the D18/Y6 binary blend. Given the high efficiency of the solar cell based on D18/Y6, it is expected that the morphology of the D18/Y6 blend is optimized, and similarly, the morphology of the D18/SM16 blend is expected to be optimized. Thus, the inefficient dissociation of the S_1 state of SM16 in the D18/SM16 blend is ascribed to a low k_{DS}.

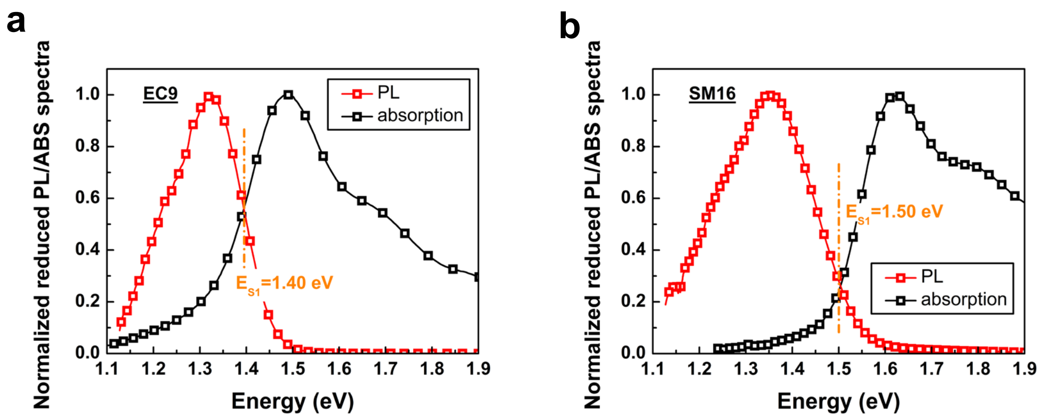

Supplementary Figure 12. Optical properties of EC9 and SM16. Normalized reduced PL and absorption spectra of **a)** the pure SM16 film and **b)** the pure EC9 film. The energy of the S_1 state (E_{S1}) is determined from the crossing point of the PL and the absorption spectra.

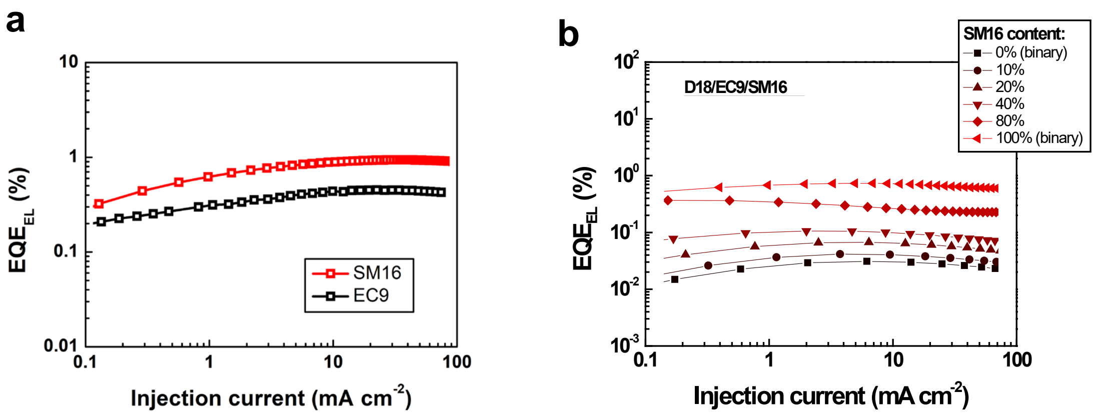

Supplementary Figure 13. Non-radiative voltage losses in ternary solar cells based on EC9 and SM16. a) EQE_{EL} of the solar cells based on pure SM16 and pure EC9 plotted as a function of injection current. **b)** EQE_{EL} of the solar cells based on D18/EC9/SM16, with different SM16 content, plotted as a function of injection current.

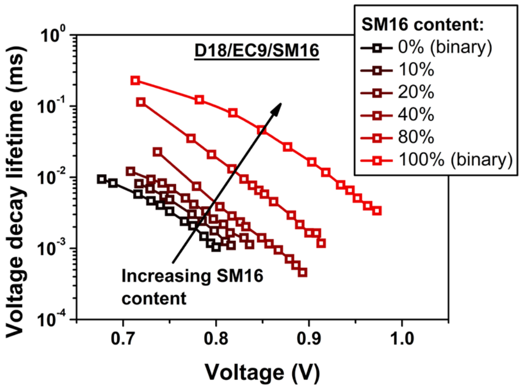

Supplementary Figure 14. Charge carrier dynamics. TPV decay lifetime of the D18/EC9/SM16 ternary solar cells with different SM16 content (see Supplementary Note 2 for details).

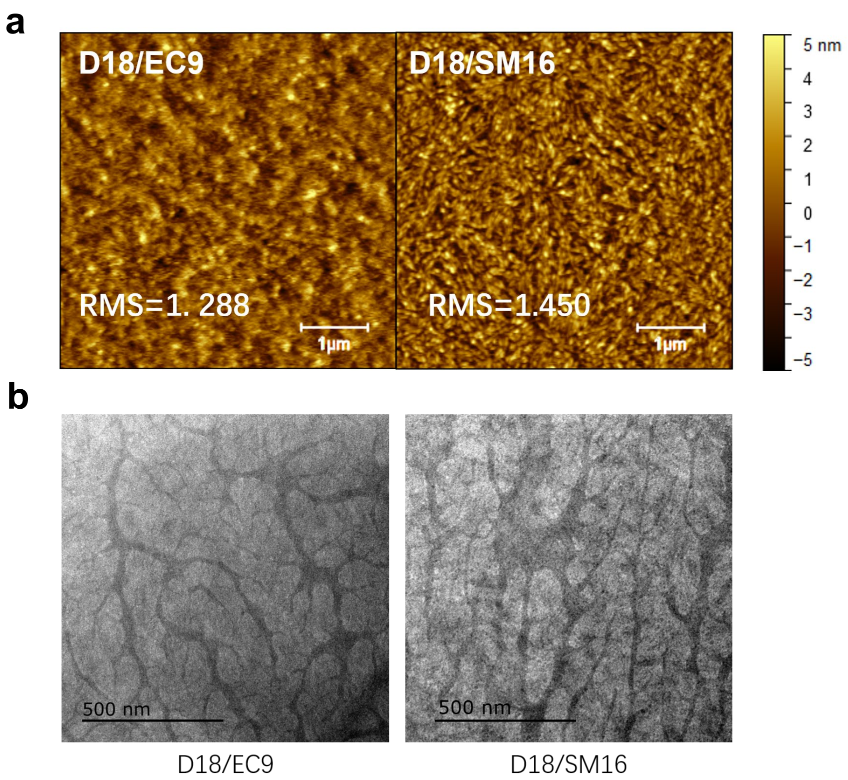

Supplementary Figure 15. Morphology characterization of the D18/EC9 and D18/SM16 blends. a) AFM and **b)** TEM of the D18/EC9 and D18/SM16 blends. The AFM and TEM images indicate that the morphologies of the active layers based on D18/EC9 and D18/SM16 are similar, and it is not expected that increasing the SM16 content in the ternary blend of D18/EC9/SM16 will result in a significant morphological change.

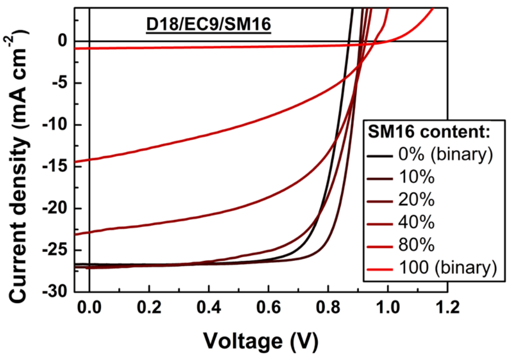

Supplementary Figure 16. Photovoltaic performance of the D18/EC9/SM16 ternary solar cells. *J*-*V* curves are recorded for the ternary solar cells with different SM16 content.

Supplementary Note 1: Derivation of analytical equations

Derivation of the expression for EQE_{EL}, assuming no decay of excited states via the triplet states

Assuming no decay of excited states via triplet states, for NFA OSCs, we can express the EQE_{EL} as

$$EQE_{EL} = \frac{n_{S_1}k_r^{S_1}+n_{CT}k_r^{CT}}{n_{S_1}k_{S_1}+n_{CT}k_{CT}} = \frac{(\frac{n_{S_1}}{n_{CT}})k_r^{S_1}+k_r^{CT}}{(\frac{n_{S_1}}{n_{CT}})k_{S_1}+k_{CT}}$$

where n_{CT} and n_{S_1} are the equilibrium concentrations of CT state and S_1^A, respectively, k_{CT} and k_{S_1} are the decay rate constants of CT state and S_1^A, respectively. Because the generation rate of S_1^A must be in equilibrium with the disappearing rate of S_1^A, we derive

$$n_{S_1}k_{S_1} + n_{S_1}k_{DS} = n_{CT}k_{BK}$$

thus,

$$\frac{n_{S_1}}{n_{CT}} = \frac{k_{BK}}{k_{S_1}+k_{DS}}$$

where k_{DS} is the dissociation rate constant, i.e., the rate constant for the transition from S_1^A to CT state, k_{BK} is the rate constant for the transition from CT state to S_1^A. Combining these equations, we have

$$EQE_{EL} = \frac{\frac{k_r^{S_1}k_{BK}}{k_{S_1}+k_{DS}}+k_r^{CT}}{\frac{k_{S_1}k_{BK}}{k_{S_1}+k_{DS}}+k_{CT}} = \frac{k_r^{S_1}k_{BK}+k_r^{CT}(k_{S_1}+k_{DS})}{k_{S_1}k_{BK}+k_{CT}(k_{S_1}+k_{DS})}$$

$$= \frac{k_r^{S_1}k_{BK}}{k_{S_1}k_{BK}+k_{CT}(k_{S_1}+k_{DS})} + \frac{k_r^{CT}(k_{S_1}+k_{DS})}{k_{S_1}k_{BK}+k_{CT}(k_{S_1}+k_{DS})}$$

$$= \frac{k_r^{S_1}}{k_{S_1}+\frac{k_{CT}(k_{S_1}+k_{DS})}{k_{BK}}} + \frac{k_r^{CT}}{\frac{k_{S_1}k_{BK}}{(k_{S_1}+k_{DS})}+k_{CT}}$$

$$= \frac{\frac{k_r^{S_1}}{k_{S_1}}}{1+\frac{k_{CT}(k_{S_1}+k_{DS})}{k_{S_1}k_{BK}}} + \frac{\frac{k_r^{CT}}{k_{CT}}}{\frac{k_{S_1}k_{BK}}{k_{CT}(k_{S_1}+k_{DS})}+1}$$

$$= \frac{\frac{k_r^{S_1}}{k_{S_1}}}{1+\frac{\frac{(k_{S_1}+k_{DS})}{k_{S_1}}}{\frac{k_{BK}}{k_{CT}}}} + \frac{\frac{k_r^{CT}}{k_{CT}}}{\frac{\frac{k_{BK}}{k_{CT}}}{\frac{k_{S_1}+k_{DS}}{k_{S_1}}}+1}$$

$$= \frac{\frac{k_r^{S_1}}{k_{S_1}}}{\left(\frac{\frac{k_{DS}}{k_{S_1}}+1}{\frac{k_{BK}}{k_{CT}}}\right)+1} + \frac{\frac{k_r^{CT}}{k_{CT}}}{\left(\frac{\frac{k_{BK}}{k_{CT}}}{\frac{k_{DS}}{k_{S_1}}+1}\right)+1}$$

Defining that

$$\eta_{S_1} = \frac{k_r^{S_1}}{k_{S_1}} \text{ and } \eta_{CT} = \frac{k_r^{CT}}{k_{CT}}$$

we can derive

$$EQE_{EL} = \frac{\eta_{S_1}}{\left(\frac{\frac{k_{DS}}{k_{S_1}}+1}{\frac{k_{BK}}{k_{CT}}}\right)+1} + \frac{\eta_{CT}}{\left(\frac{\frac{k_{BK}}{k_{CT}}}{\frac{k_{DS}}{k_{S_1}}+1}\right)+1}$$

Then, we define that

$$r_{DS} = \frac{k_{DS}}{k_{S_1}} \quad \text{and} \quad r_{BK} = \frac{k_{BK}}{k_{CT}}$$

and finally, we derive

$$EQE_{EL} = \frac{\eta_{S_1}}{\left(\frac{r_{DS}+1}{r_{BK}}\right)+1} + \frac{\eta_{CT}}{\left(\frac{r_{BK}}{r_{DS}+1}\right)+1}$$

Derivation of the expression for EQE_{EL} in the case of the excited states partially decaying via triplet states

In case of having non-geminate decay of excited states via triplet states, for NFA OSCs, we can express the EQE_{EL} as

$$EQE_{EL} = \frac{n_{S_1}k_r^{S_1} + n_{CT_1}k_r^{CT_1}}{n_{S_1}k_{S_1} + n_{CT_1}k_{CT_1} + n_{CS}k_{CS}^{CT_3}}$$

where n_{CT_1}, n_{S_1}, and n_{CS} are the equilibrium concentrations of singlet CT state, S_1^A, and charge separated state, respectively, k_{CT_1} and k_{S_1} are the decay rate constants of singlet CT state and S_1^A, respectively. $k_{CS}^{CT_3}$ is the rate constant for the transfer of charge separated state to the triplet CT state. Here, we assume that the excited states are directly lost upon forming the triplet CT states from charge-separated states. This assumption is based on the expectation that the conversion of triplet CT states to the molecular triplet states (T_1) is much faster than the conversion of triplet CT states to the singlet CT states or the dissociation of triplet CT states into free charge carriers (*Nature* 2021, 597, 666). A schematic illustration of the excited state dynamics for NFA OSCs under EL or EQE_{EL} measurements is presented below in Supplementary Figure 17.

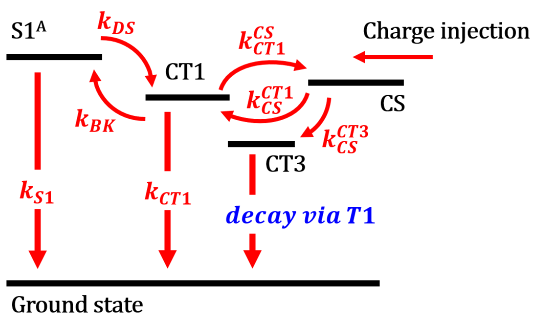

Supplementary Figure 17. Excited states dynamics. Excited states in NFA OSCs under EL or EQE_{EL} measurement in the case of the excited states partially decaying via triplet states. S_1^A, CT_1, CT_3, and CS are abbreviations for acceptor singlet, singlet charge transfer, triplet charge transfer, and charge separated states, respectively.

Assuming a simple spin statistical approximation, 75% of free charge carrier recombine via CT_3, we have

$$n_{CS}k_{CS}^{CT_3} = 3n_{CS}k_{CS}^{CT_1}$$

where $k_{CS}^{CT_1}$ is the rate constant for the transfer of charge separated state to the singlet CT state. Then, the expression for EQE_{EL} becomes,

$$EQE_{EL} = \frac{n_{S_1} k_r^{S_1} + n_{CT_1} k_r^{CT_1}}{n_{S_1} k_{S_1} + n_{CT_1} k_{CT_1} + 3 n_{CS} k_{CS}^{CT_1}}$$

Because the generation rate of CT_1 must be in equilibrium with the disappearing rate of CT_1, we derive

$$n_{CT_1}\left(k_{BK} + k_{CT_1}^{CS} + k_{CT_1}\right) = n_{S_1} k_{DS} + n_{CS} k_{CS}^{CT_1}$$

where k_{BK} is the rate constant for the transfer of CT_1 to S_1^A, $k_{CT_1}^{CS}$ is the rate constant for the dissociation of CT_1 into free charge carriers, k_{CT_1} is the rate constant for the decay of CT_1 to the ground state, and k_{DS} is the rate constant of the dissociation of S_1^A. This gives

$$n_{CS} k_{CS}^{CT_1} = n_{CT_1}\left(k_{BK} + k_{CT_1}^{CS} + k_{CT_1}\right) - n_{S_1} k_{DS}$$

Then, the expression for EQE_{EL} becomes

$$EQE_{EL} = \frac{n_{S_1} k_r^{S_1} + n_{CT_1} k_r^{CT_1}}{n_{S_1} k_{S_1} + n_{CT_1} k_{CT_1} + 3 n_{CT_1}\left(k_{BK} + k_{CT_1}^{CS} + k_{CT_1}\right) - 3 n_{S_1} k_{DS}}$$

$$= \frac{(\frac{n_{S_1}}{n_{CT_1}}) k_r^{S_1} + k_r^{CT_1}}{(\frac{n_{S_1}}{n_{CT_1}}) k_{S_1} + k_{CT_1} + 3\left(k_{BK} + k_{CT_1}^{CS} + k_{CT_1}\right) - 3(\frac{n_{S_1}}{n_{CT_1}}) k_{DS}}$$

Since the generation rate of S_1^A must be in equilibrium with the disappearing rate of S_1^A, we derive

$$n_{S_1}(k_{S_1} + k_{DS}) = n_{CT_1} k_{BK}$$

Thus,

$$\frac{n_{S_1}}{n_{CT_1}} = \frac{k_{BK}}{k_{S_1} + k_{DS}}$$

Combining the above two equations, we have

$$EQE_{EL} = \frac{\left(\frac{k_{BK}}{k_{S_1} + k_{DS}}\right) k_r^{S_1} + k_r^{CT_1}}{\left(\frac{k_{BK}}{k_{S_1} + k_{DS}}\right) k_{S_1} + k_{CT_1} + 3\left(k_{BK} + k_{CT_1}^{CS} + k_{CT_1}\right) - 3\left(\frac{k_{BK}}{k_{S_1} + k_{DS}}\right) k_{DS}}$$

$$= \frac{k_{BK} k_r^{S_1} + k_r^{CT_1}\left(k_{S_1} + k_{DS}\right)}{k_{BK} k_{S_1} + k_{CT_1}\left(k_{S_1} + k_{DS}\right) + 3\left(k_{BK} + k_{CT_1}^{CS} + k_{CT_1}\right)\left(k_{S_1} + k_{DS}\right) - 3 k_{BK} k_{DS}}$$

$$
= \frac{k_{BK} k_r^{S_1}}{k_{BK} k_{S_1} + k_{CT_1}\left(k_{S_1} + k_{DS}\right) + 3\left(k_{BK} + k_{CT_1}^{CS} + k_{CT_1}\right)\left(k_{S_1} + k_{DS}\right) - 3k_{BK}k_{DS}}
$$

$$
+ \frac{k_r^{CT_1}\left(k_{S_1} + k_{DS}\right)}{k_{BK} k_{S_1} + k_{CT_1}\left(k_{S_1} + k_{DS}\right) + 3\left(k_{BK} + k_{CT_1}^{CS} + k_{CT_1}\right)\left(k_{S_1} + k_{DS}\right) - 3k_{BK}k_{DS}}
$$

$$
= \frac{k_r^{S_1}}{k_{S_1} + \frac{k_{CT_1}\left(k_{S_1} + k_{DS}\right) + 3\left(k_{BK} + k_{CT_1}^{CS} + k_{CT_1}\right)\left(k_{S_1} + k_{DS}\right) - 3k_{BK}k_{DS}}{k_{BK}}}
$$

$$
+ \frac{k_r^{CT_1}}{k_{CT_1} + \frac{k_{BK} k_{S_1} + 3\left(k_{BK} + k_{CT_1}^{CS} + k_{CT_1}\right)\left(k_{S_1} + k_{DS}\right) - 3k_{BK}k_{DS}}{\left(k_{S_1} + k_{DS}\right)}}
$$

Defining that

$$
\eta_{S_1} = \frac{k_r^{S_1}}{k_{S_1}} \quad \text{and} \quad \eta_{CT_1} = \frac{k_r^{CT_1}}{k_{CT_1}}
$$

we derive

$$
EQE_{EL} = \frac{\eta_{S_1}}{\frac{k_{CT_1}\left(k_{S_1} + k_{DS}\right) + 3\left(k_{BK} + k_{CT_1}^{CS} + k_{CT_1}\right)\left(k_{S_1} + k_{DS}\right) - 3k_{BK}k_{DS}}{k_{S_1}k_{BK}} + 1}
$$

$$
+ \frac{\eta_{CT_1}}{\frac{k_{BK} k_{S_1} + 3\left(k_{BK} + k_{CT_1}^{CS} + k_{CT_1}\right)\left(k_{S_1} + k_{DS}\right) - 3k_{BK}k_{DS}}{k_{CT_1}\left(k_{S_1} + k_{DS}\right)} + 1}
$$

$$
= \frac{\eta_{S_1}}{\frac{k_{CT_1}k_{S_1} + k_{CT_1}k_{DS} + 3k_{BK}k_{S_1} + 3k_{CT_1}^{CS}k_{S_1} + 3k_{CT_1}k_{S_1} + 3k_{BK}k_{DS} + 3k_{CT_1}^{CS}k_{DS} + 3k_{CT_1}k_{DS} - 3k_{BK}k_{DS}}{k_{S_1}k_{BK}} + 1}
$$

$$
+ \frac{\eta_{CT1}}{\frac{k_{BK}k_{S_1} + 3k_{BK}k_{S_1} + 3k_{CT_1}^{CS}k_{S_1} + 3k_{CT_1}k_{S_1} + 3k_{BK}k_{DS} + 3k_{CT_1}^{CS}k_{DS} + 3k_{CT_1}k_{DS} - 3k_{BK}k_{DS}}{k_{CT_1}\left(k_{S_1} + k_{DS}\right)} + 1}
$$

$$
= \frac{\eta_{S_1}}{\frac{\left(\frac{k_{DS}}{k_{S_1}} + 1\right)}{\frac{k_{BK}}{k_{CT_1}}} \cdot \left(4 + 3\frac{k_{CT_1}^{CS}}{k_{CT_1}}\right) + 4} + \frac{\eta_{CT_1}}{\frac{\frac{k_{BK}}{k_{CT_1}}}{\left(\frac{k_{DS}}{k_{S_1}} + 1\right)} \cdot 4 + (4 + 3\frac{k_{CT_1}^{CS}}{k_{CT_1}})}
$$

Finally, defining that

$$
r_{DS} = \frac{k_{DS}}{k_{S_1}}, \quad r_{BK} = \frac{k_{BK}}{k_{CT_1}}, \text{ and } P = 4 + 3\frac{k_{CT_1}^{CS}}{k_{CT_1}}
$$

we have

$$EQE_{EL} = \frac{\eta_{S_1}}{\frac{(r_{DS}+1)}{r_{BK}} \cdot P + 4} + \frac{\eta_{CT}}{\frac{r_{BK}}{(r_{DS}+1)} \cdot 4 + P}$$

The expression derived in the case of the excited state partially decaying via triplet states is similar to that derived assuming no decay of excited states via triplet states. The main difference lies in the addition of a positive parameter P (>4) in the denominators of the expression. This term is determined by the ratio between the rate constant for the dissociation of singlet CT state into free charge carriers ($k_{CT_1}^{CS}$) and the rate constant for the direct decay of singlet CT state (k_{CT_1}).

Then, we ran computer simulations using the expression for EQE_{EL} mentioned above and equation (7) in the main text of the article to illustrate the impact of r_{DS} and r_{BK} on the performance of solar cells in the case of the excited state partially decaying via triplet states. As depicted in Supplementary Figure 18, both EQE_{EL} and PCE are lower in the solar cell with excited states decaying via triplet states compared to that with no decay via triplet states, even when the P value is at its minimum of 4. However, regardless of the P value, we observed an increase in EQE_{EL} and PCE with the reduction of k_{DS}. Therefore, the conclusion that EQE_{EL} increases with the reduction of k_{DS} remains valid, in both cases with or without excited states decaying via triplet states.

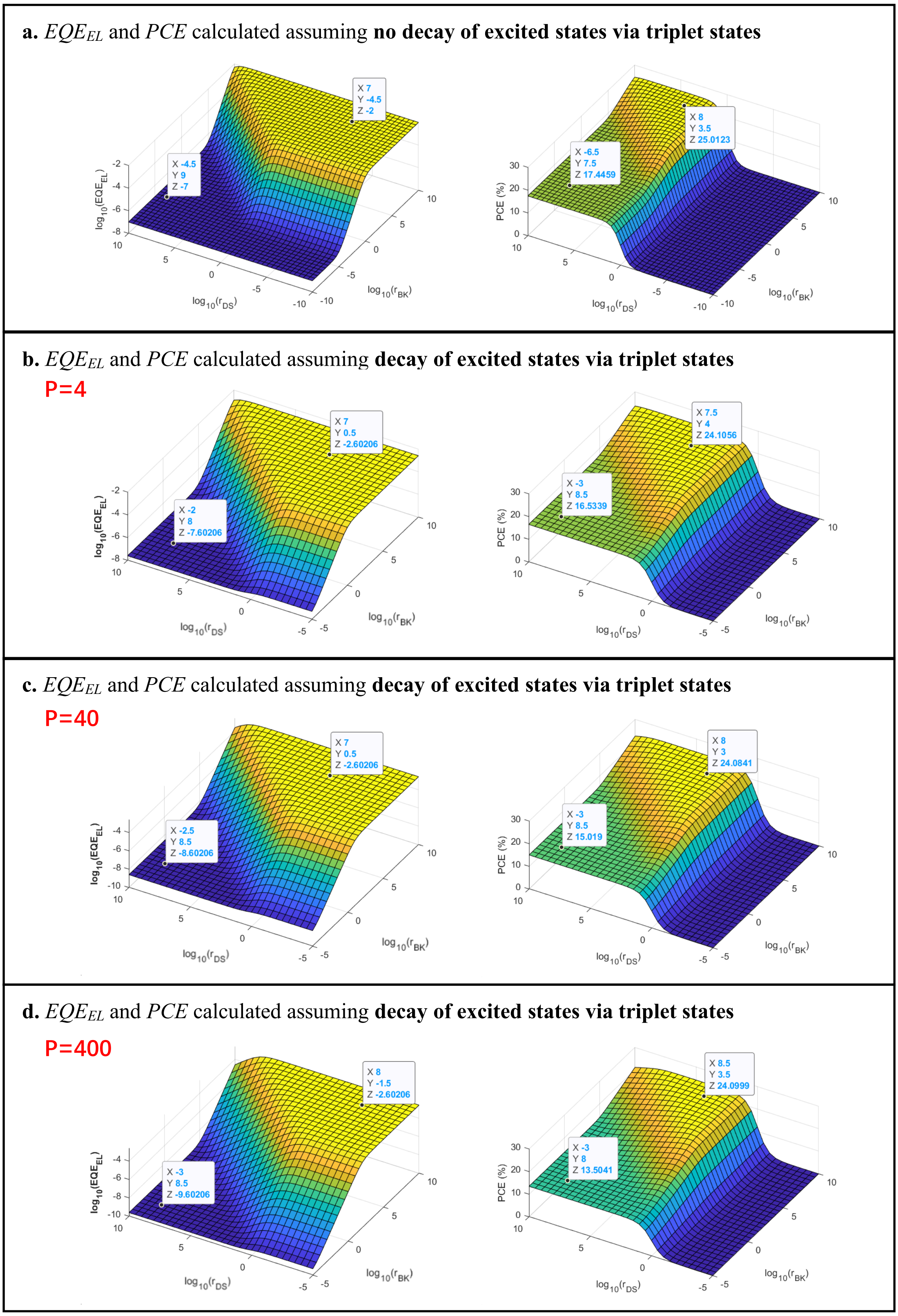

Supplementary Figure 18. Simulation results. EQE_{EL} and PCE as a function of r_{DS} and r_{BK}, **a)** calculated using equation (5) and equation (7) in the main text, and calculated using the expression for EQE_{EL} derived above and equation (7) in the main text, by assuming **b)** P=4, **c)** P=40, and **d)** P=400. η_{S_1} and η_{CT_1} of the solar cell are assumed to be 1% and 10^{-5}%, respectively. The radiative limit for V_{OC}, the upper limit for J_{SC}, and the FF of the solar cell are assumed to be 1.10 V, 32 mA cm^{-2}, and 80%, respectively.

Derivation of the expression for *IQE*

The excited state dynamics of an NFA OSC under short circuit and constant illumination is illustrated below in Supplementary Figure 19, in which k_{DS}^{CT} is the dissociation rate constant of CT state, k_{BK}^{SC} is the rate constant for free charge carriers to form CT state at the D/A interface, k_{CT} and k_{S_1} are the decay rate constants of CT state and S_1^A, respectively. k_{DS} is the dissociation rate constant, i.e., the rate constant for the transition from S_1^A to CT state, k_{BK} is the rate constant for the transition from CT state to S_1^A.

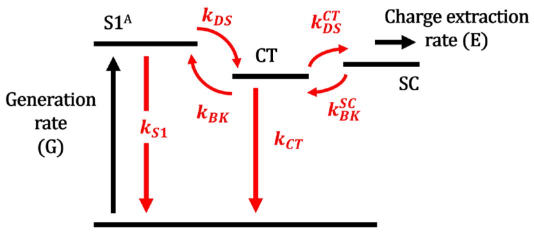

Supplementary Figure 19. Excited states dynamics for the short circuit condition. Excited states in NFA OSCs under short circuit and continuous illumination, where S_1^A, CT, SC, are the abbreviations for acceptor singlet, charge transfer, and charge separated states, respectively.

The *IQE* of the solar cell can be expressed as

$$IQE = \frac{E}{G}$$

where *E* is the extract rate of charge carriers, and *G* is the generation rate of S_1 state. Assuming that under short-circuit, the built-in field in the solar cell prevents back transfer of CT state, recombination of CT state, and formation of CT state from the charge separated state, then,

$$E = n_{CT}k_{DS}^{CT} - n_{SC}k_{BK}^{SC}$$

where n_{CT} and n_{SC} are the equilibrium concentrations of CT state and free charge carriers. Since the generation rate of CT state must be in equilibrium with the disappearing rate of CT state,

$$n_{S_1}k_{DS} + n_{SC}k_{BK}^{SC} = n_{CT}k_{BK} + n_{CT}k_{CT} + n_{CT}k_{DS}^{CT}$$

where n_{S_1} is the concentration of S_1^A, we derive

$$E = n_{S_1}k_{DS} - n_{CT}(k_{BK} + k_{CT})$$

Since the generation rate of S_1^A must be in equilibrium with the dissociation rate and decay rate of S_1^A, and the back transfer rate and the decay rate of CT state, we have

$$G = n_{S_1}(k_{S_1} + k_{DS}) - n_{CT}k_{BK}$$

Thus,

$$IQE = \frac{n_{S_1}k_{DS} - n_{CT}(k_{BK}+k_{CT})}{n_{S_1}(k_{S_1}+k_{DS}) - n_{CT}k_{BK}} = \frac{k_{DS} - \left(\frac{n_{CT}}{n_{S_1}}\right)k_{BK} - \left(\frac{n_{CT}}{n_{S_1}}\right)k_{CT}}{(k_{S_1}+k_{DS}) - \left(\frac{n_{CT}}{n_{S_1}}\right)k_{BK}}$$

Assuming that under short-circuit, n_{CT} is much smaller than n_{S_1}, due to the high built-in electric field in the solar cell,

$$\frac{n_{CT}}{n_{S_1}} \approx 0$$

Thus, we derive

$$IQE \approx \frac{k_{DS}}{k_{S_1}+k_{DS}} = \frac{r_{DS}}{r_{DS}+1}$$

Supplementary Note 2: Details about field dependent PL measurements

The lower k_{DS} of the PM7/Y5 blend, compared to that of the PM7/IT4F blend, as well as the reduction of k_{DS} of the ternary PM7/IT4F/Y5 blend, with increasing Y5 content, can also be deduced from the field dependent steady state PL results.

Specifically, for the PM7/IT4F solar cell, the steady-state PL is significantly quenched compared to the PL emission from the S_1 state of neat IT4F (Supplementary Figure 20a). This suggests a homogeneous mixture between PM7 and IT4F in the blend, allowing for the diffusion of the S_1 state to a donor/acceptor interface within the lifetime of the S_1 state. It also suggests a fast dissociation of the S_1 state at the donor/acceptor interfaces. The PL intensity of the PM7/IT4F solar cell is hardly dependent on the electric field (Supplementary Figure 20b) because all S_1 states can dissociate efficiently without an electric field. Therefore, the charge generation yield in PM7/IT4F is high, leading to a high EQE_{PV}.

For the PM7/Y5 solar cell, the steady-state PL emission is not significantly quenched compared to that of the neat Y5 film (Supplementary Figure 20a). This suggests inefficient dissociation of the S_1 state in the PM7/Y5 blend. This inefficiency could be attributed either to a too large phase separation between PM7 and Y5, preventing excitons from diffusing to a donor/acceptor interface within the lifetime of S_1 states, or to a too low dissociation rate of the S_1 states at the donor/acceptor interface. In the first case, the emission is not expected to be electric field-dependent, as the diffusion of the S_1 state is not field-dependent. However, electric field-dependent PL measurements for the PM7/Y5 solar cell (Supplementary Figure 20c) revealed a significant quenching of the PL intensity with increasing electric field. This indicates that the dissociation of the S_1 states at PM7/Y5 interfaces is indeed slow but facilitated by the electric field. In fact, the PL signal is quenched by 80% with a sufficiently large applied field, implying that the diffusion of excitons or the morphology of the active layer is not a severe issue for the PM7/Y5 blend. Therefore, the charge generation yield in PM7/Y5 is limited by the dissociation rate of the S_1 state, leading to a low EQE_{PV}.

For the ternary PM7/IT4F/Y5 solar cell with a Y5 content of 10%, emission peaks from S_1 states of both IT4F and Y5 are observed in the steady-state PL spectra (Supplementary Figure 20d). Strikingly, we note that the field dependency of both IT4F and Y5 emissions in the ternary blend is stronger than that of the IT4F emission in the PM7/IT4F binary blend. This indicates that the addition of a small amount of Y5 could lead to a reduction in the dissociation rate of the S_1 state in the ternary blend. However, this reduction in dissociation rate does not notably affect the charge generation yield, as confirmed by the steady-state PL measurements: The quenching efficiency of the ternary blend with a Y5 content of 10% is similar to that of the binary PM7/IT4F blend (Supplementary Figure 20a). Therefore, the EQE_{PV} of the ternary solar cell with a Y5 content of 10% is high, close to that of the binary PM7/IT4F solar cell.

For the ternary PM7/IT4F/Y5 solar cell with an 80% Y5 content, the steady-state PL emission is not significantly quenched compared to that of the neat Y5 film (Supplementary Figure 20a), similar to

what was observed for the binary PM7/Y5 solar cell. This suggests an inefficient dissociation of the S_1 state in the ternary PM7/IT4F/Y5 solar cell with an 80% Y5 content. Electric field-dependent PL measurements for the ternary PM7/IT4F/Y5 solar cell with an 80% Y5 content (Supplementary Figure 20e) revealed a significant quenching of the PL intensity with increasing electric field, indicating that the inefficient dissociation of the S_1 state originates from the slow dissociation of the S_1 states at donor/acceptor interfaces. Therefore, the charge generation yield in the ternary PM7/IT4F/Y5 solar cell with an 80% Y5 content is limited by the dissociation rate of the S_1 state, leading to a low EQE_{PV}.

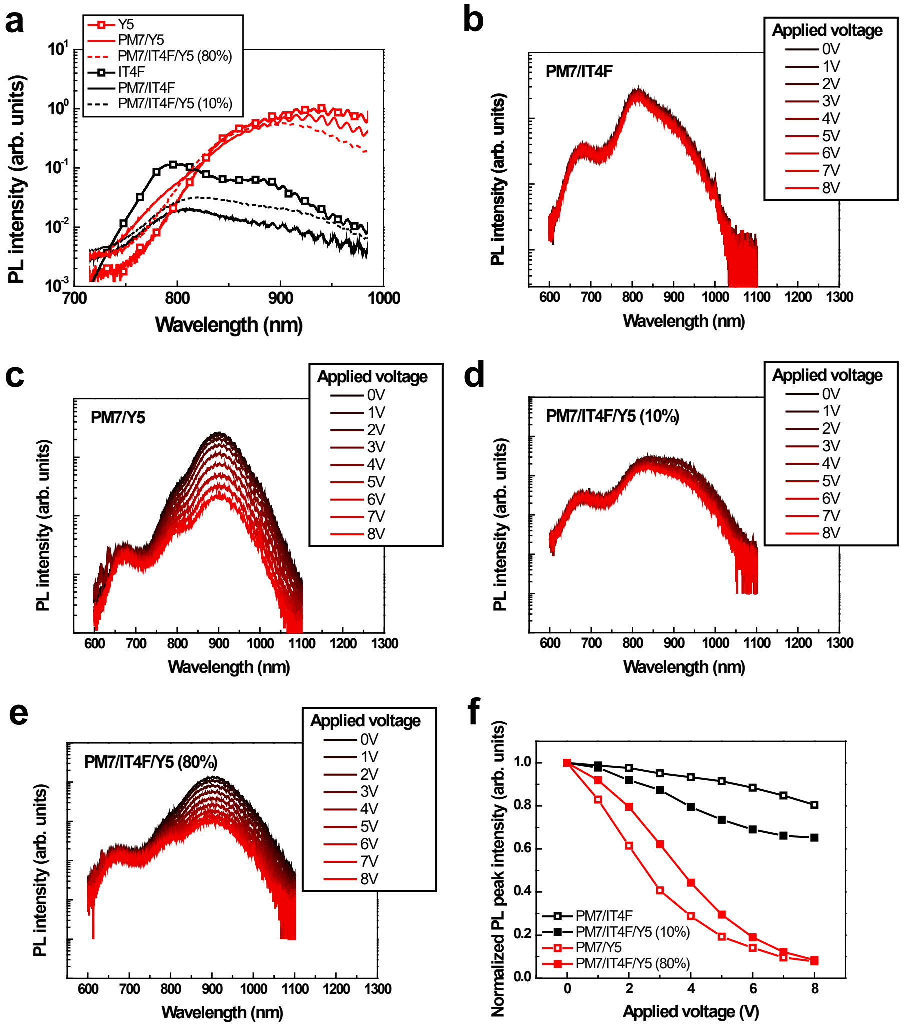

Supplementary Figure 20. Photoluminescence of the PM7/IT4F/Y5 systems. a) Steady state PL spectra of the PM7/IT4F and PM7/Y5 binary blends, the IT4F and Y5 pure films, and the ternary PM7/IT4F/Y5 (10%) and PM7/IT4F/Y5 (80%) films. Electric field dependent PL spectra of the solar cells based on **b)** PM7/IT4F, **c)** PM7/Y5, **d)** PM7/IT4F/Y5 (10%), and **e)** PM7/IT4F/Y5 (80%). **f)** PL peak intensity as a function of applied voltage for solar cells based on PM7/IT4F, PM7/Y5, PM7/IT4F/Y5 (10%), and PM7/IT4F/Y5 (80%).

Supplementary Note 3: Details about TPV measurements

Transient photovoltage decay measurement

Transient Photovoltage (TPV) measurements were conducted using an LED lamp as the background light source, powered by a Keithley 2400 for bias illumination. Additionally, another LED, controlled by an arbitrary wave generator (Tektronix, AFG3022C), was employed to deliver pulsed illumination, with a time interval between consecutive pulses maintained under 5 milliseconds (ms). Transient photovoltage signals were captured using an oscilloscope (MDO4104C, Tektronix). Specifically, the intensity of the background light from the bias LED was adjusted to enable the generation of photovoltage in the organic solar cells close to the V_{OC} value determined by *J-V* characterization. Subsequently, pulsed light was introduced, creating an additional transient photovoltage in the device. The intensity of the pulsed light was carefully adjusted to ensure that the additional transient photovoltaic is approximately 5% of the bias photovoltage. By gradually reducing the intensity of both the bias and pulsed illumination, decay signals were measured for devices at different photovoltages. These signals were then fitted by an exponential decay function to determine the lifetime of the transient photovoltage. Below, we provide the recorded decay signals for the organic solar cells under various bias illumination intensities, along with the fitting curves used to determine voltage decay lifetimes.

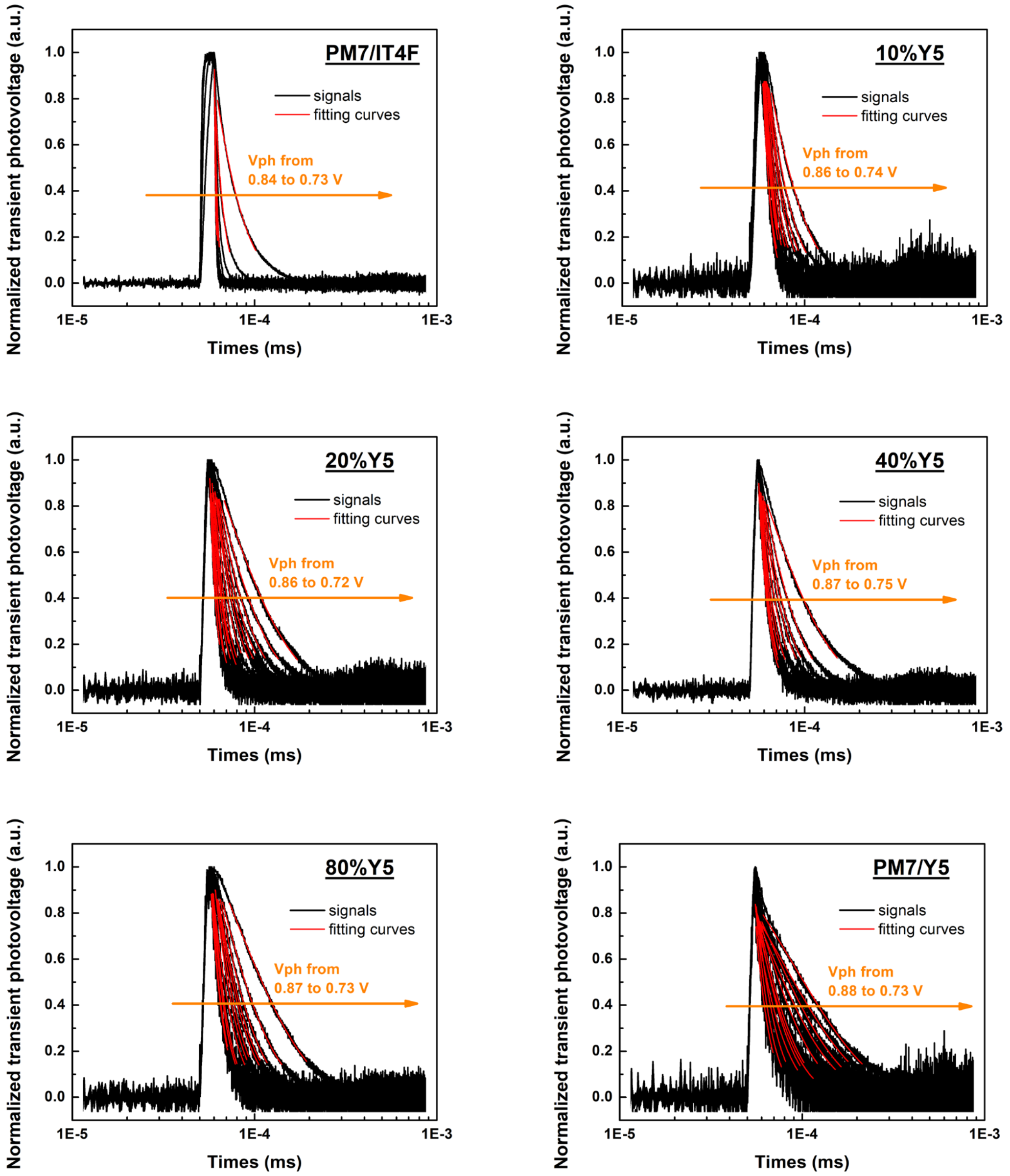

Supplementary Figure 21. Transient photovoltage decay measurements for the PM7/IT4F/Y5 systems. Normalized transient photovoltage decay signals (black) and fitting curves (red) for the binary solar cells based on PM7/IT4F and PM7/Y5, and the ternary solar cells with different Y5 content.

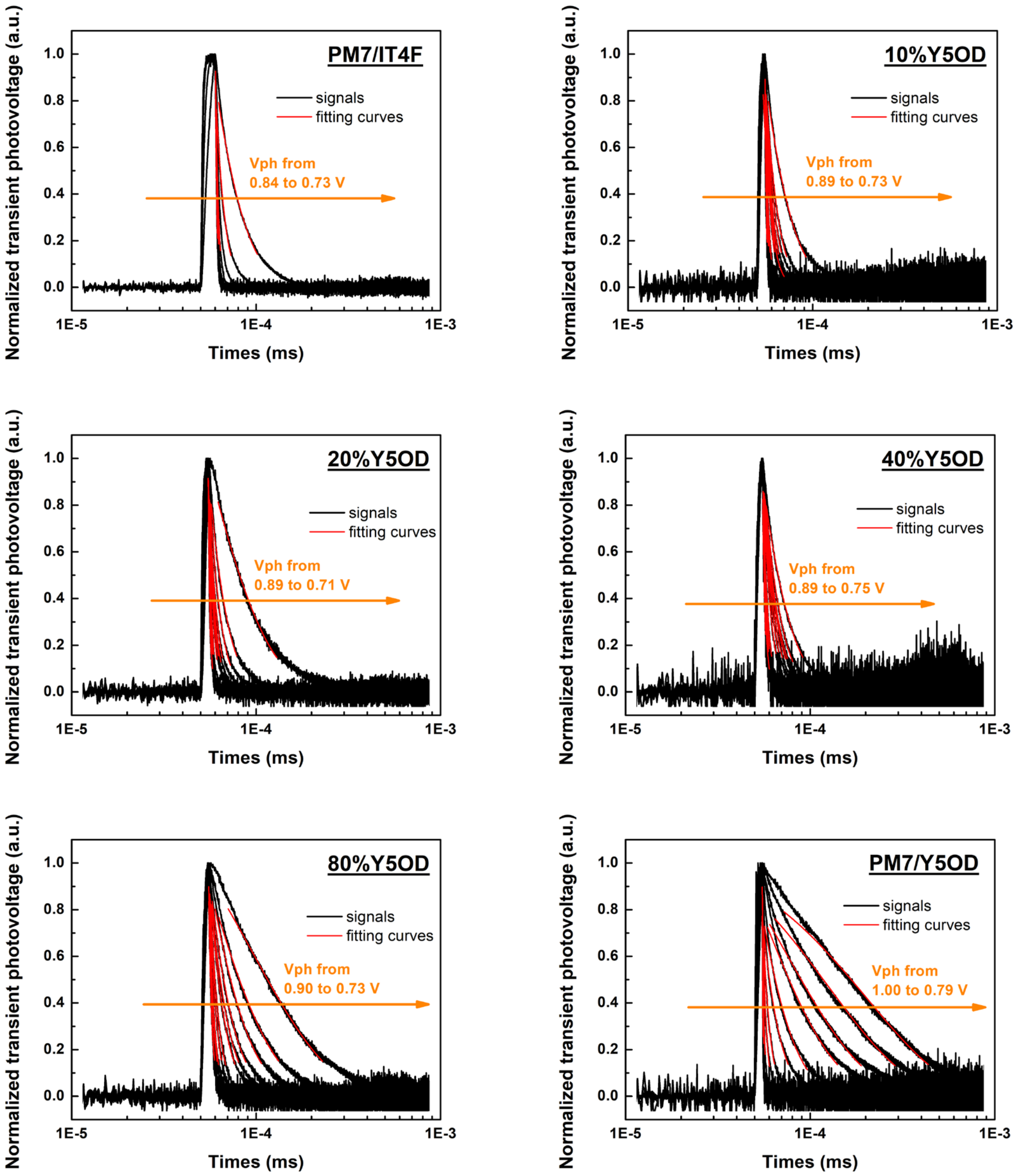

Supplementary Figure 22. Transient photovoltage decay measurements for the PM7/IT4F/Y5OD systems. Normalized transient photovoltage decay signals (black) and fitting curves (red) for the binary solar cells based on PM7/IT4F and PM7/Y5OD, and the ternary solar cells with different Y5OD content.

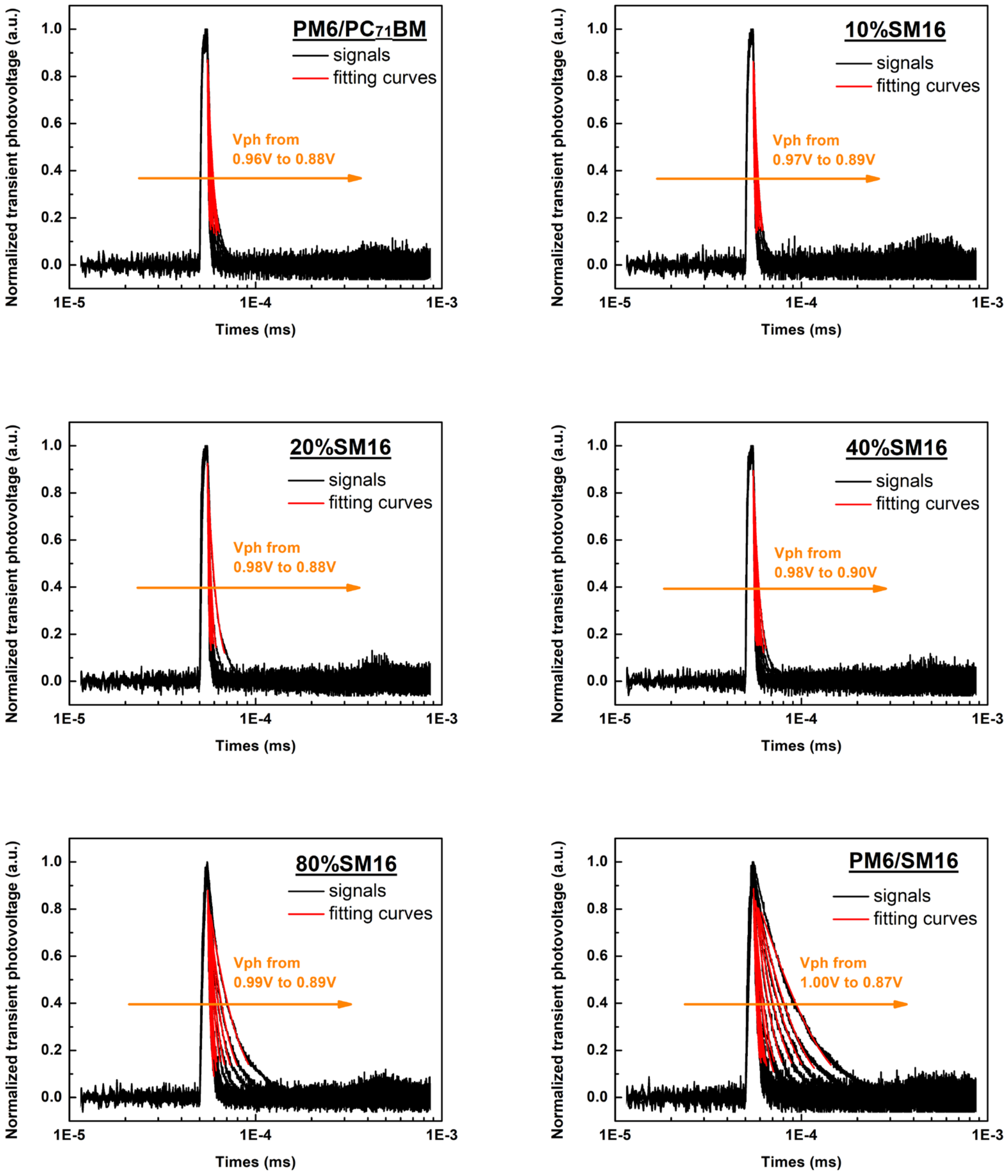

Supplementary Figure 23. Transient photovoltage decay measurements for the PM6/PC$_{71}$BM/SM16 systems. Normalized transient photovoltage decay signals (black) and fitting curves (red) for the binary solar cells based on PM6/PC$_{71}$BM and PM6/SM16, and the ternary solar cells with different SM16 content.

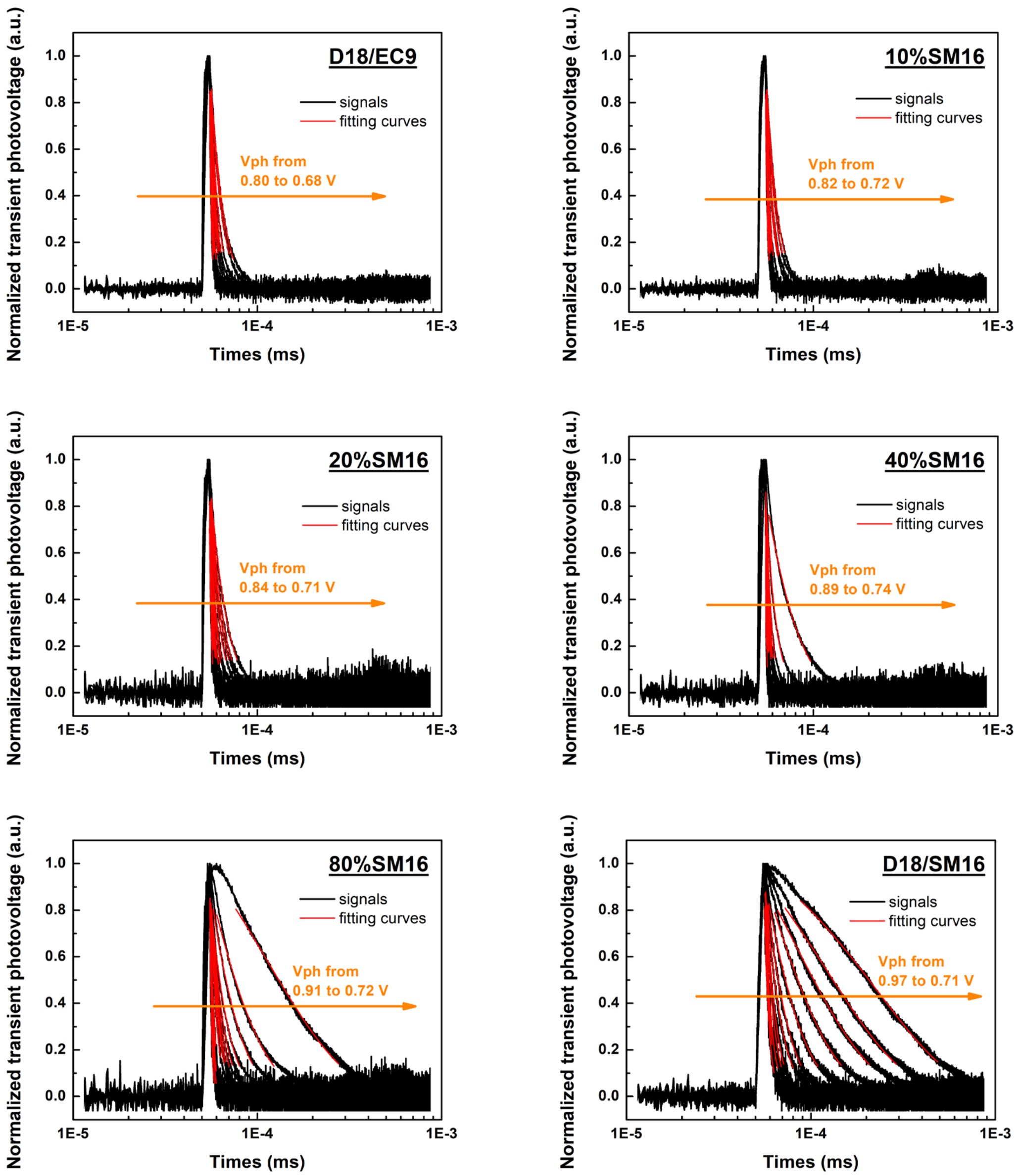

Supplementary Figure 24. Transient photovoltage decay measurements for the D18/EC9/SM16 systems. Normalized transient photovoltage decay signals (black) and fitting curves (red) for the binary solar cells based on D18/EC9 and D18/SM16, and the ternary solar cells with different SM16 content.

Supplementary Note 4: Ternary solar cells based on PM7/IT4F/Y5OD

In this work, we have also employed Y5OD (chemical structure shown in Supplementary Figure 1) as the secondary acceptor for the PM7/IT4F blend to evaluate the effectiveness of the dual acceptor strategy for improving the performance of OSCs. From the EL spectrum of the PM7/Y5OD binary solar cell, we only resolve the emission from the S_1 state of Y5OD (Supplementary Figure 25a), implying that ΔE_{CT} of the PM7/Y5OD blend is low, and k_{DS} is also low. Because of the low k_{DS}, exciton dissociation efficiency in the PM7/Y5OD binary solar cell is severely limited, as confirmed by the PL measurements (Supplementary Figure 25b). Accordingly, *IQE* of the PM7/Y5OD binary solar cell is low, leading to low peak EQE_{PV}, about 20% (Supplementary Figure 25c). The S_1 state energy of Y5OD (1.46 eV, Supplementary Figure 25d) is very close to E_{CT} of the PM7/IT4F blend (1.46 eV, Supplementary Figure 2). Besides, the emission efficiencies of the S_1 states of Y5OD (0.5%) and IT4F (0.2%) are not very different (Supplementary Figure 25e). Therefore, in the PM7/IT4F/Y5OD ternary blend, k_{DS} is expected to decrease with the increasing Y5OD content.

Because of the decrease in k_{DS}, the dominant peak in the EL spectrum (Supplementary Figure 25a) of the PM7/IT4F/Y5OD ternary solar cell changes from the PM7/IT4F CT state emission peak to the Y5OD S_1 state emission peak, with the increasing Y5OD content, and the device EQE_{EL} significantly increases, as shown in Supplementary Figure 26a: EQE_{EL} of the ternary solar cell with a Y5OD content of 10% is increased by over an order of magnitude, compared to that of the PM7/IT4F binary solar cell. Accordingly, the lifetime of charge carriers in the PM7/IT4F/Y5OD ternary solar cell is increased with the increased Y5OD content (Supplementary Figure 26b), and V_{OC} is also increased, as shown in Supplementary Figure 26a. Meanwhile, EQE_{PV} does not decrease with the addition of a small amount of Y5OD: The peak EQE_{PV} of the solar cell with a Y5OD content of 10% is very close to that of the PM7/IT4F binary solar cell (Supplementary Figure 25c). Since the *FF* of the ternary solar cell is not much affected by the addition of a small amount of Y5OD (Supplementary Table 1), *PCE* of the solar cell is increased from 11.8% to 13.8%, when the Y5OD content is increased from 0% to 20% (Supplementary Figure 26c). The basic photovoltaic performance parameters of the solar cells with different Y5OD content are summarized in Supplementary Table 1, and the *J-V* curves are provided in Supplementary Figure 26d.

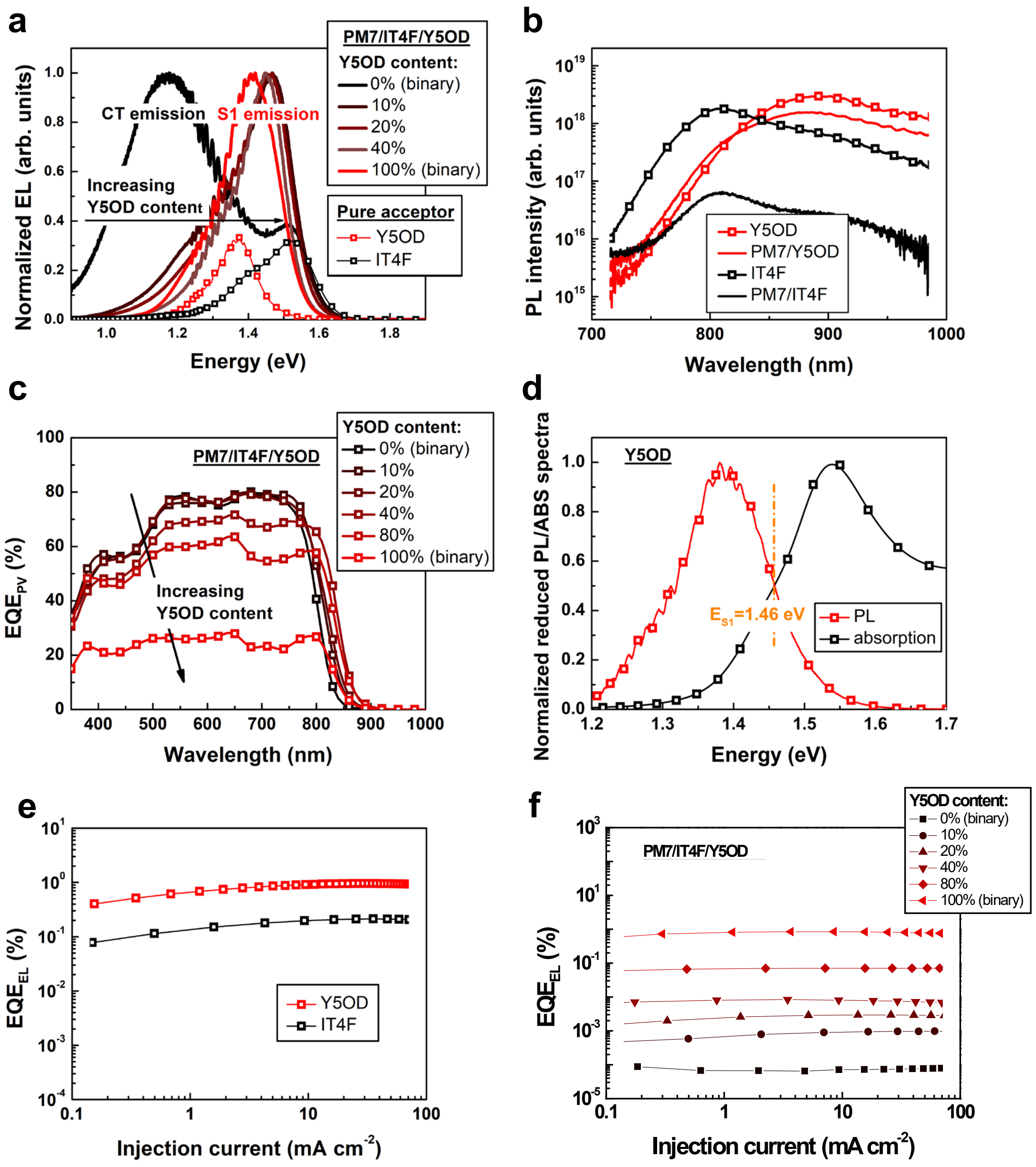

Supplementary Figure 25. Characterization of the PM7/IT4F/Y5OD ternary blends. a) EL spectra of the solar cells based on the binary blends of PM7/IT4F and PM7/Y5OD, and the ternary blends of PM7/IT4F/Y5OD with different Y5OD content, and the EL spectra of the devices based on pure IT4F and pure Y5OD. **b)** PL spectra of the PM7/IT4F and PM7/Y5OD binary blends, and the IT4F and Y5OD pure films. The excitation wavelength used is 500 nm. **c)** EQE_{PV} of the PM7/IT4F/Y5OD ternary solar cells with different Y5OD content. **d)** Normalized reduced PL and absorption spectra of the pure Y5OD film. The energy of the S_1 state (E_{S1}) of Y5OD is determined from the crossing point of the PL and the absorption spectra. **e)** EQE_{EL} of the devices based on pure IT4F and pure Y5OD plotted as a function of injection current. **f)** EQE_{EL} of the solar cells based on PM7/IT4F/Y5OD, with different Y5OD content, plotted as a function of injection current.

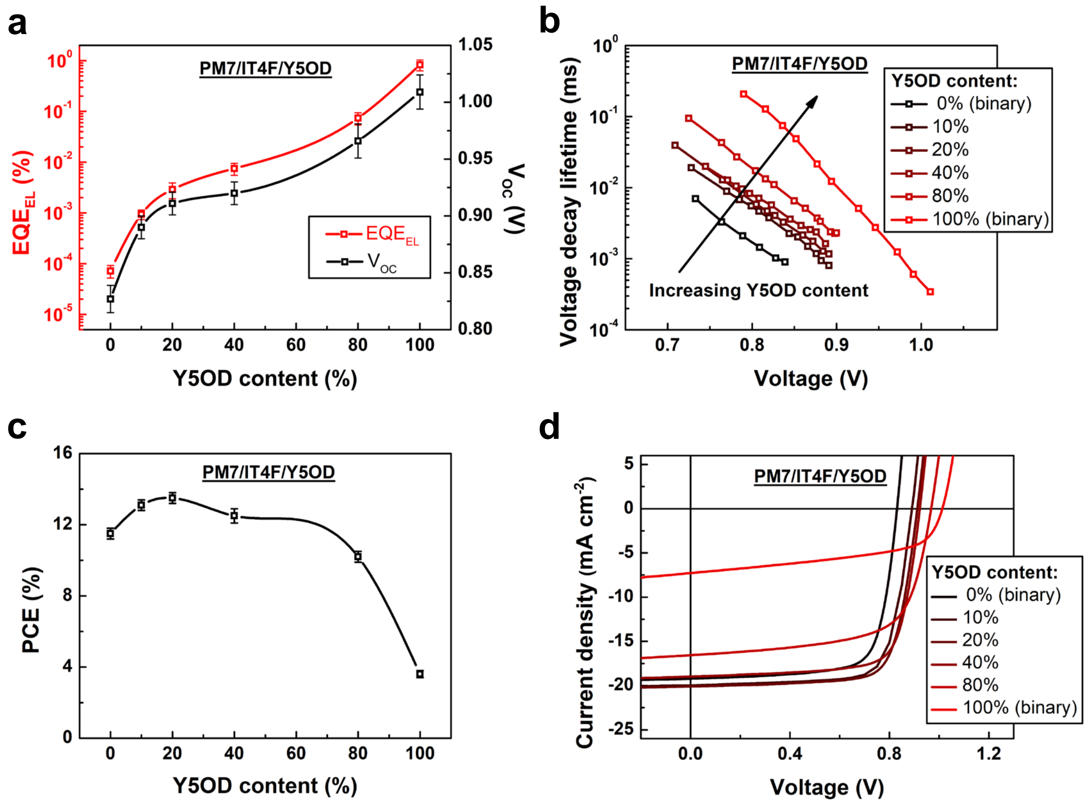

Supplementary Figure 26. Characterization of the PM7/IT4F/Y5OD ternary solar cells. a) EQE_{EL} and V_{OC} of PM7/IT4F/Y5OD ternary solar cells with different Y5OD content. **b)** TPV decay lifetime of the PM7/IT4F/Y5OD ternary solar cells with different Y5OD content. **c)** *PCE* of the PM7/IT4F/Y5OD ternary solar cells with different Y5OD content. **d)** *J-V* curves of the PM7/IT4F/Y5OD ternary solar cells with different Y5OD content. The error bars in **a)** and **c)** represent the highest, lowest, and average values from multiple devices.

Supplementary Table 1. Representative performance parameters of the solar cells based on PM7/IT4F/Y5OD, with different Y5OD content. The *J-V* curves of the devices are provided in Supplementary Figure 26d. The statistic results are obtained from 8 individual devices.

Y5OD content	V_{OC} (V)	J_{SC} (mA cm^{-2})	FF (%)	PCE (%)	EQE_{EL} (%)	V_{NR} (eV)
0%	0.827 (0.820±0.012)	19.2 (19.1±0.3)	74.4 (74.0±1.2)	11.8 (11.5±0.3)	7.2×10^{-5}	0.354
10%	0.890 (0.880±0.013)	20.0 (19.9±0.2)	75.1 (74.8±1.0)	13.4 (13.1±0.3)	9.4×10^{-4}	0.289
20%	0.911 (0.900±0.012)	20.1 (20.0±0.2)	75.2 (75.0±1.1)	13.8 (13.5±0.3)	2.9×10^{-3}	0.261
40%	0.920 (0.910±0.013)	19.0 (18.8±0.3)	74.0 (73.5±1.0)	12.9 (12.5±0.4)	7.5×10^{-3}	0.237
80%	0.966 (0.953±0.016)	16.6 (16.5±0.2)	65.5 (65.0±1.1)	10.5 (10.2±0.3)	7.4×10^{-2}	0.180
100%	1.009 (1.000±0.015)	7.2 (7.1±0.2)	52.8 (52.3±1.2)	3.8 (3.6±0.2)	8.3×10^{-1}	0.120

Supplementary Note 5: Ternary solar cells based on PM6/ fullerene/SM16

We have additionally employed SM16 (chemical structure shown in Supplementary Figure 1) as the secondary acceptor for the PM6/$PC_{71}BM$ blend to evaluate the effectiveness of the dual acceptor strategy for improving the performance of OSCs. From the EL spectrum of the PM6/SM16 binary solar cell, we only resolve the emission from the S_1 state of SM16 (Supplementary Figure 27a), implying that ΔE_{CT} of the PM6/SM16 blend is low, and k_{DS} is also low. Because of the low k_{DS}, exciton dissociation efficiency in the PM6/SM16 binary solar cell is severely limited, as confirmed by the PL measurements (Supplementary Figure 27b). Accordingly, *IQE* of the PM6/SM16 binary solar cell is low, leading to low peak EQE_{PV}, about 10% (Supplementary Figure 27c). The S_1 state energy of SM16 (1.50 eV, Supplementary Figure 27d) is comparable to the E_{CT} of the PM6/$PC_{71}BM$ blend (1.57 eV, Supplementary Figure 27e). Furthermore, the emission efficiencies of the S_1 states of $PC_{71}BM$ (0.01%) is lower than that of SM16 (0.9%) (Supplementary Figure 27f). Accordingly, in the PM6/$PC_{71}BM$/SM16 ternary blend, k_{DS} is expected to decrease with the increasing SM16 content.

Because of the decrease in k_{DS}, the dominant peak in the EL spectrum (Supplementary Figure 27a) of the PM6/$PC_{71}BM$/SM16 ternary solar cell changes from the PM6/$PC_{71}BM$ CT state emission peak to the SM16 S_1 state emission peak, with the increasing SM16 content, and the device EQE_{EL} significantly increases, as shown in Supplementary Figure 28a: EQE_{EL} of the ternary solar cell with a SM16 content of 10% is increased by over an order of magnitude, compared to that of the PM6/$PC_{71}BM$ binary solar cell. Accordingly, the lifetime of charge carriers in the PM6/$PC_{71}BM$/SM16 ternary solar cell is increased with the increased SM16 content (Supplementary Figure 28b), and V_{OC} is also increased, as shown in Supplementary Figure 28a. Meanwhile, EQE_{PV} does not decrease with the addition of a small amount of SM16: The peak EQE_{PV} of the solar cell with a SM16 content of 10% is close to that of the PM6/$PC_{71}BM$ binary solar cell (Supplementary Figure 27c). Since the *FF* of the ternary solar cell is not reduced by the addition of a small amount of SM16 (Supplementary Table 2), *PCE* of the solar cell is increased from 6.48% to 7.81%, when the SM16 content is increased from 0% to 20% (Supplementary Figure 28c). The basic photovoltaic performance parameters of the solar cells with different SM16 content are summarized in Supplementary Table 2, and the *J-V* curves are provided in Supplementary Figure 28d.

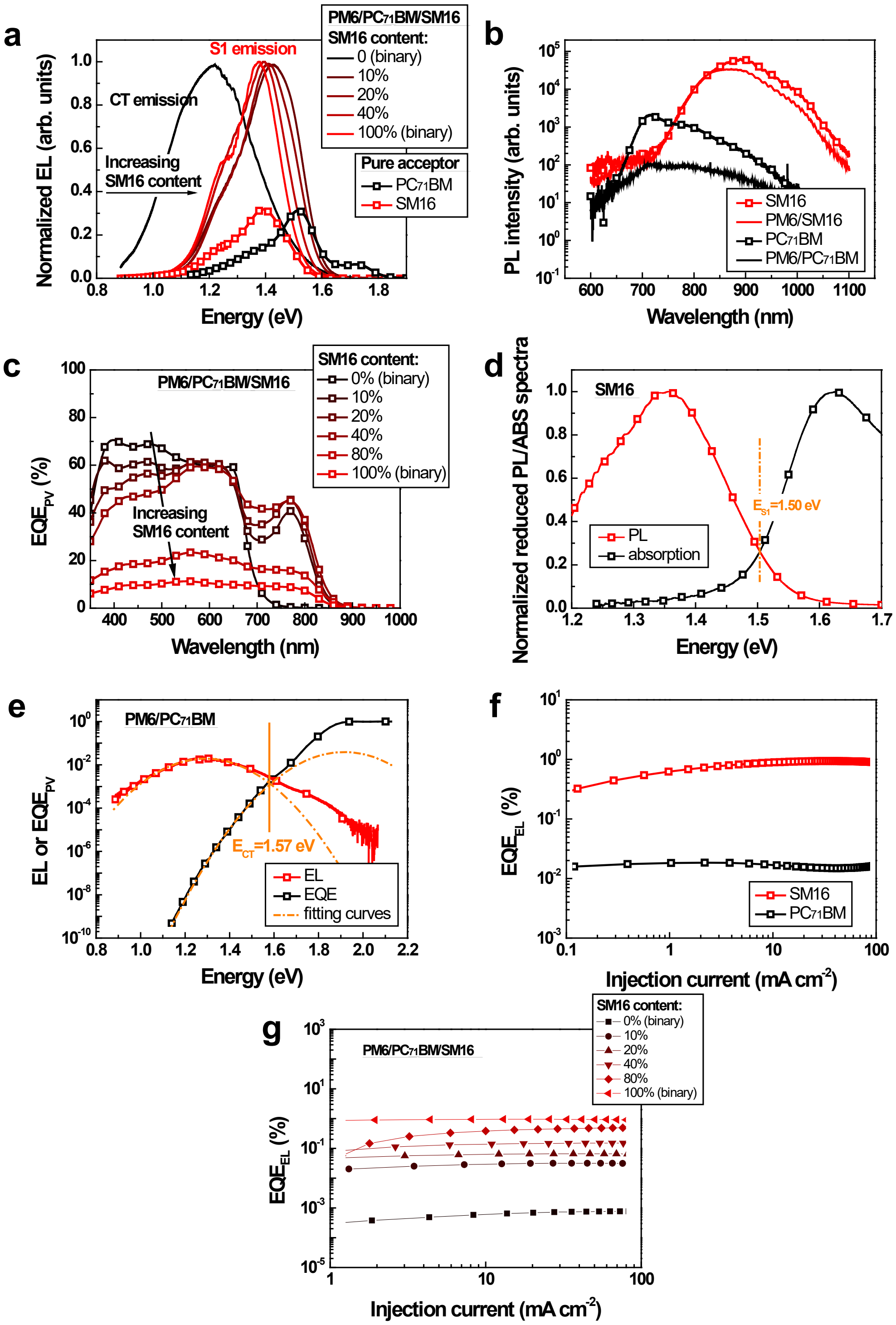

Supplementary Figure 27. Characterization of the PM6/PC$_{71}$BM/SM16 ternary blend. a) EL spectra of the solar cells based on the binary blends of PM6/PC$_{71}$BM and PM6/SM16, and the ternary blends of PM6/PC$_{71}$BM/SM16 with different SM16 content, and the EL spectra of the devices based on pure PC$_{71}$BM and pure SM16. **b)** PL spectra of the PM6/PC$_{71}$BM and PM6/SM16 binary blends, and the PC$_{71}$BM and SM16 pure films. The excitation wavelength used is 500 nm. **c)** EQE_{PV} of the PM6/PC$_{71}$BM/SM16 ternary solar cells with different SM16 content. **d)** Normalized reduced PL and absorption spectra of the pure SM16 film. The energy of the S$_1$ state (E_{S1}) of SM16 is determined from the crossing point of the PL and the absorption spectra. **e)** Sensitive EL and EQE_{PV} spectra of the PM6/PC$_{71}$BM solar cell. E_{CT} is determined by a Gaussian fitting to the lower energy part of the EQE_{PV} spectrum using the method described in the literature (Phys. Rev. B 81, 125204 (2010)). **f)** EQE_{EL} of the devices based on pure PC$_{71}$BM and pure SM16, plotted as a function of injection current. **g)** EQE_{EL} of the solar cells based on PM6/PC$_{71}$BM/SM16, with different SM16 content, plotted as a function of injection current.

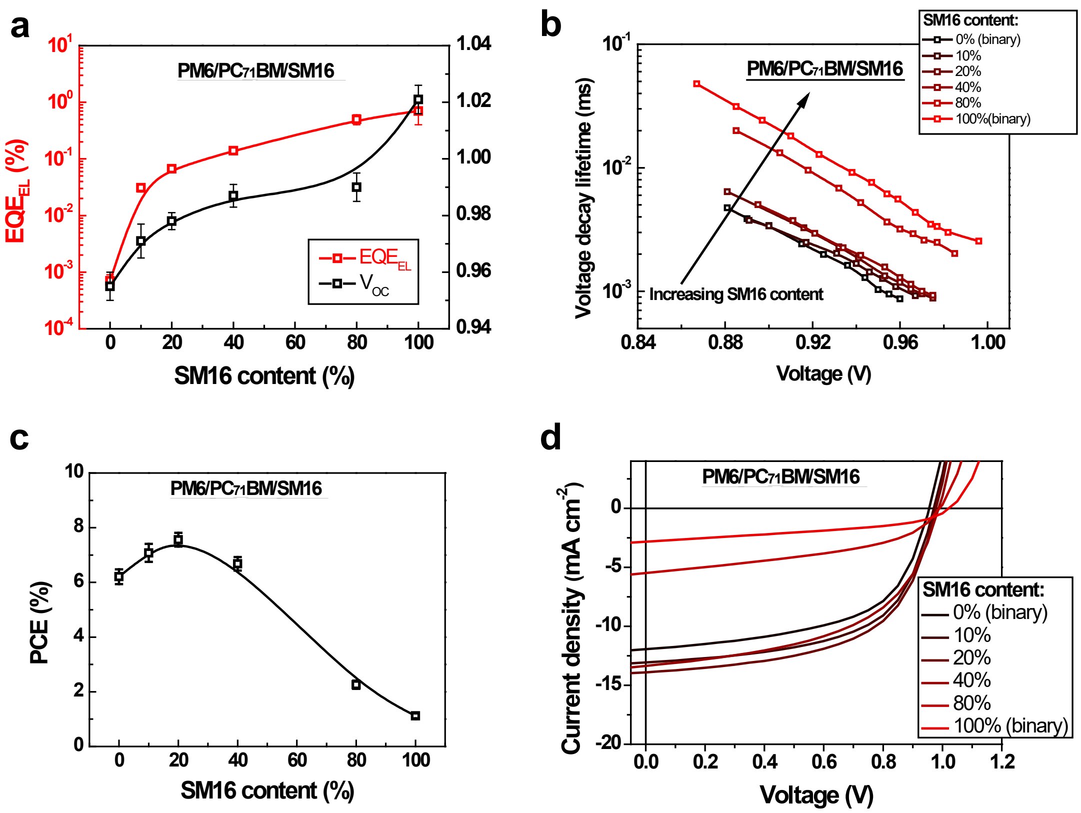

Supplementary Figure 28. Characterization of the PM6/PC$_{71}$BM/SM16 ternary solar cells. a) EQE_{EL} and V_{OC} of PM6/PC$_{71}$BM/SM16 ternary solar cells with different SM16 content. **b)** TPV decay lifetime of the PM6/PC$_{71}$BM/SM16 ternary solar cells with different SM16 content. **c)** *PCE* of the PM6/PC$_{71}$BM/SM16 ternary solar cells with different SM16 content. **d)** *J-V* curves of the PM6/PC$_{71}$BM/SM16 ternary solar cells with different SM16 content. The error bars in **a)** and **c)** represent the highest, lowest, and average values from multiple devices.

Supplementary Table 2. Representative performance parameters of the solar cells based on PM6/PC$_{71}$BM/SM16, with different SM16 content. The *J-V* curves of the devices are provided in Supplementary Figure 28d. The statistic results are obtained from 8 individual devices.

SM16 content	V_{OC} (V)	J_{SC} (mA cm^{-2})	*FF* (%)	*PCE* (%)	EQE_{EL} (%)	V_{NR} (eV)
0%	0.955 (0.950±0.005)	11.9 (11.7±0.2)	56.9 (55.9±1.0)	6.48 (6.21±0.27)	7.0×10^{-4}	0.297
10%	0.971 (0.965±0.006)	13.1 (12.8±0.3)	58.4 (57.3±1.1)	7.41 (7.08±0.33)	3.1×10^{-2}	0.202
20%	0.978 (0.975±0.003)	13.9 (13.8±0.1)	57.4 (56.5±0.9)	7.81 (7.56±0.25)	6.7×10^{-2}	0.183
40%	0.987 (0.983±0.004)	13.4 (13.2±0.2)	52.6 (51.5±1.1)	6.93 (6.68±0.25)	1.4×10^{-1}	0.164
80%	0.990 (0.985±0.005)	5.5 (5.3±0.2)	44.3 (43.3±1.0)	2.41 (2.26±0.15)	5.0×10^{-1}	0.132
100%	1.021 (1.016±0.005)	2.8 (2.7±0.1)	41.8 (40.7±1.1)	1.21 (1.12±0.09)	1.0×10^{0}	0.115

Supplementary Note 6: Ternary solar cells based on PM7/IT4F/BTA3

In Case 2 of Figure 2 in the main text, it can be deduced that using A2 with a S_1 state energy (E_{S1}) higher than that of A1 could also result in an increased E_{CT} and reduced k_{CT} in the ternary blend as the A2 content is increased. In this scenario, the employment of A2 is expected to significantly enhance EQE_{EL}, as A2 with a higher E_{S1} is often more emissive than A1. Therefore, we employed BTA3 (chemical structure shown in Supplementary Figure 1) with a high E_{S1} value of 1.84 eV (Supplementary Figure 29a) as the secondary acceptor for the PM7/IT4F blend to assess the possibility of achieving a more effective increase in EQE_{EL} and a more significant enhancement in solar cell performance.

From the EL spectrum of the PM7/BTA3 binary solar cell, we only resolve the emission from the S_1 state of BTA3 (Supplementary Figure 29b), implying that ΔE_{CT} of the PM7/BTA3 blend is low, and k_{DS} is also low. Because of the low k_{DS}, exciton dissociation efficiency in the PM7/BTA3 binary solar cell is severely limited, thus, *IQE* is low, leading to low peak EQE_{PV}, only about 30% (Supplementary Figure 29c).

However, we note that the PM7/IT4F CT state emission peak dominates the EL spectrum (Supplementary Figure 29b) of the PM7/IT4F/BTA3 ternary solar cell, and increasing the BTA3 content only leads to increased emission intensity of the S_1 state of IT4F. This suggests that there is energy transfer between the S_1 states of BTA3 and IT4F in the ternary blend. Then, we perform PL measurements for the blend of BTA3 and IT4F, and the pure BTA3 and IT4F films. As shown in Supplementary Figure 29d, we find that the PL emission of the BTA3/IT4F blend, excited at the wavelength where both BTA3 and IT4F strongly absorb (500 nm), is completely dominated by the emission from IT4F, despite that the S_1 state of BTA3 is more emissive than that of IT4F (Supplementary Figure 29e). This confirms that there is indeed energy transfer between BTA3 and IT4F.

Because of energy transfer, the use of BTA3 has little impact on k_{DS} of the ternary blend, and EQE_{EL} of the ternary solar cell hardly increases with the BTA3 content (Supplementary Figure 29f). Since *IQE* and *FF* of the ternary solar cell decrease with the increasing BTA3 content, *PCE* of the solar cell is reduced. The basic photovoltaic performance parameters of the solar cells with different BTA3 content are summarized in Supplementary Table 3, and the *J-V* curves are provided in Supplementary Figure 30a. The *PCE* of OSCs with the different BTA3 content are provided in Supplementary Figure 30b.

The above results indicate that the use of the secondary acceptor with the S_1 state energy higher than the primary D/A1 blend would have limited impact on k_{DS}, due to energy transfer between the secondary and the primary acceptor. Therefore, it would not result in an increase in EQE_{EL}, or improved performance of the solar cell.

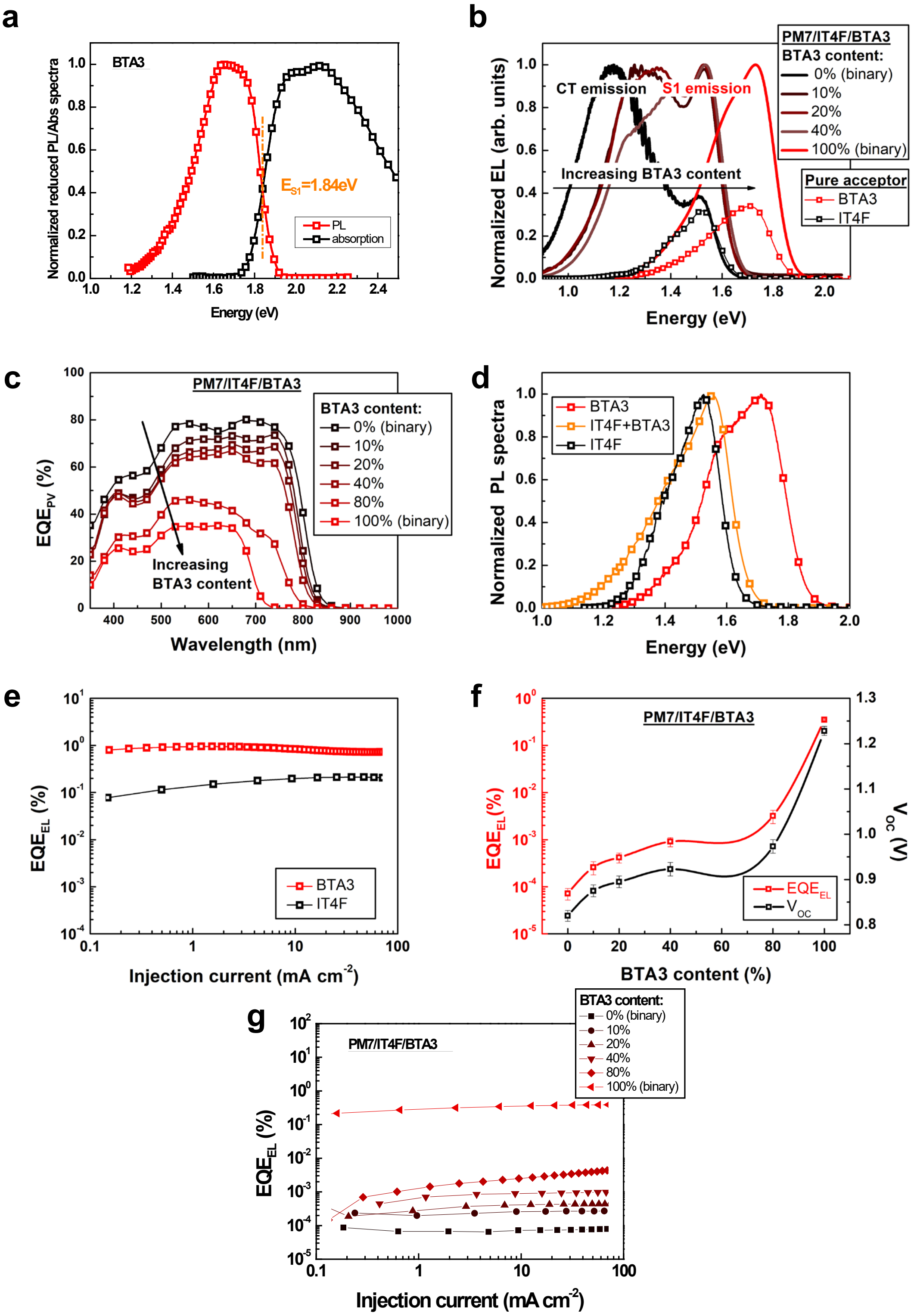

Supplementary Figure 29. Characterization of the PM7/IT4F/BTA3 ternary blend. a) Normalized reduced PL and absorption spectra of the pure BTA3 film. The energy of the S_1 state (E_{S1}) of BTA3 is determined from the crossing point of the PL and the absorption spectra. **b)** EL spectra of the solar cells based on the binary blends of PM7/IT4F and PM7/BTA3, and the ternary blends of PM7/IT4F/BTA3 with different BTA3 content, and the EL spectra of the devices based on pure IT4F and pure BTA3. **c)** EQE_{PV} of the PM7/IT4F/BTA3 ternary solar cells with different BTA3 content. **d)** PL spectra of the pure BTA3, pure IT4F films, and the blend BTA3/IT4F film. **e)** EQE_{EL} of the devices based on pure BTA3 and pure IT4F plotted as a function of injection current. **f)** EQE_{EL} and V_{OC} of the PM7/IT4F/BTA3 ternary solar cells with different BTA3 content. **g)** EQE_{EL} of the solar cells based on PM7/IT4F/BTA3, with different BTA3 content, plotted as a function of injection current. The error bars represent the highest, lowest, and average values from multiple devices.

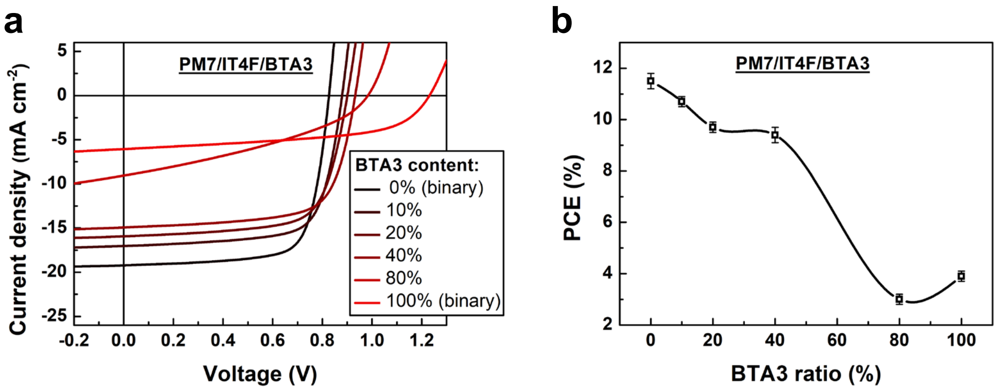

Supplementary Figure 30. Characterization of the PM7/IT4F/BTA3 ternary blend. a) *J-V* curves of OSCs with increasing content of BTA3. **b)** *PCE* of OSCs with different BTA3 content. The error bars represent the highest, lowest, and average values from multiple devices.

Supplementary Table 3. Representative performance parameters of the solar cells based on PM7/IT4F/BTA3. The statistic results are obtained from 8 individual devices.

BTA3 content	V_{OC} (V)	J_{SC} (mA cm^{-2})	*FF* (%)	*PCE* (%)	EQE_{EL} (%)	V_{NR} (eV)
0%	0.827 (0.820±0.012)	19.2 (19.1±0.3)	74.4 (74.0±1.2)	11.8 (11.5±0.3)	7.2×10^{-5}	0.354
10%	0.883 (0.875±0.013)	17.0 (16.9±0.2)	72.7 (72.0±1.1)	10.9 (10.7±0.2)	2.6×10^{-4}	0.322
20%	0.903 (0.895±0.013)	15.9 (15.8±0.2)	69.8 (69.0±1.0)	10.0 (9.7±0.3)	4.2×10^{-4}	0.310
40%	0.933 (0.923±0.015)	14.9 (14.8±0.1)	69.4 (69.0±1.1)	9.7 (9.4±0.3)	9.1×10^{-4}	0.290
80%	0.985 (0.973±0.015)	9.1 (9.0±0.2)	36.3 (36.0±1.0)	3.2 (3.0±0.2)	3.2×10^{-3}	0.259
100%	1.232 (1.228±0.010)	6.1 (6.1±0.2)	54.7 (54.0±1.3)	4.1 (3.9±0.2)	3.5×10^{-1}	0.141
