# Supplementary material for: Decreasing exciton dissociation rates for reduced voltage losses in organic solar cells
Source: Nat Commun. 2024 Mar 27;15:2693. doi: 10.1038/s41467-024-46797-5 (PMC10973424; doi:10.1038/s41467-024-46797-5)
Supplement: Supplementary file 3 — Reporting Summary [file 41467_2024_46797_MOESM3_ESM.pdf]

nature portfolio

Corresponding author(s): Zaifei Ma, Zhishan Bo, Zheng Tang

Solar Cells Reporting Summary

Nature Portfolio wishes to improve the reproducibility of the work that we publish. This form is intended for publication with all accepted papers reporting the characterization of photovoltaic devices and provides structure for consistency and transparency in reporting. Some list items might not apply to an individual manuscript, but all fields must be completed for clarity.

For further information on Nature Research policies, including our data availability policy, see Authors & Referees.

Experimental design

Please check the following details are reported in the manuscript, and provide a brief description or explanation where applicable.

1. Dimensions

Item	Yes/No	Response
Area of the tested solar cells	☒ Yes ☐ No	Active area is 0.04 cm2
		Explain why this information is not reported/not relevant.
Method used to determine the device area	☒ Yes ☐ No	Device area was calibrated using an optical microscope. Details are provided in Methods.
		Explain why this information is not reported/not relevant.

2. Current-voltage characterization

Item	Yes/No	Response
Current density-voltage (J-V) plots in both forward and backward direction	☐ Yes ☒ No	No difference observed for the JV curves measured in forward and backward directions.
Voltage scan conditions	☐ Yes ☒ No	*Provide a description of the measurement conditions (e.g. scan direction, speed, dwell times).*
		No difference observed for the JV curves measured with different scan conditions.
Test environment	☒ Yes ☐ No	The measurements were done under an ambient condition.
		Explain why this information is not reported/not relevant.
Protocol for preconditioning of the device before its characterization	☐ Yes ☒ No	*Provide a description of the protocol.*
		No preconditioning needed for the characterization of the devices
Stability of the J-V characteristic	☐ Yes ☒ No	*Provide a description of the method used. The stability of the J-V characteristic can be verified with time evolution of the maximum power point or with the photocurrent at maximum power point; see ref. 5 for details.*
		The devices studied in this work are based on a standard device structure and commonly used active materials systems Stability studies on organic solar cells can be found in the literature.

3. Hysteresis or any other unusual behaviour

Item	Yes/No	Response
Description of the unusual behaviour observed during the characterization	☐ Yes ☒ No	*Provide a description of hysteresis or any other unusual behaviour observed during the characterization.*
		No unusual behavior observed during the measurement.
Related experimental data	☐ Yes ☒ No	*Provide a description of the related experimental data.*
		No unusual behavior observed during the measurement.

4. Efficiency

Item	Yes/No	Response
External quantum efficiency (EQE) or incident photons to current efficiency (IPCE)	☒ Yes ☐ No	EQE measurements were done under a strong bias illumination, using a halogen lamp, a monochromator, a lock-in amplifier, and a preamplifier. Details are provided in Methods.
		Explain why this information is not reported/not relevant.

A comparison between the integrated response under the standard reference spectrum and the response measure under the simulator

☒ Yes Details are provided in Methods.
☐ No *Explain why this information is not reported/not relevant.*

For tandem solar cells, the bias illumination and bias voltage used for each subcell

☐ Yes *Provide a description of the measurement conditions.*
☒ No No tandem devices studied in this work.

5. Calibration

Light source and reference cell or sensor used for the characterization

☒ Yes The light source is an Newport AAA solar simulator calibrated by a standard silicon solar cell and a set of low pass filters.
☐ No *Explain why this information is not reported/not relevant.*

Confirmation that the reference cell was calibrated and certified

☒ Yes The reference cell was calibrated by external quantum efficiency measurements (under a bias illumination intensity of 100 mW cm-2).
☐ No *Explain why this information is not reported/not relevant.*

Calculation of spectral mismatch between the reference cell and the devices under test

☒ Yes Spectral mismatch was calibrated by using a reference cell and a set of low-pass optical filters.
☐ No *Explain why this information is not reported/not relevant.*

6. Mask/aperture

Size of the mask/aperture used during testing

☒ Yes 0.02 cm2
☐ No *Explain why this information is not reported/not relevant.*

Variation of the measured short-circuit current density with the mask/aperture area

☐ Yes *Report the difference in the short-circuit current density values measured with the mask and aperture area.*
☒ No The reported short-circuit current density values were compared to the current density derived from the sensitive EQE measurements (measured with a bias illumination intensity of 100 mW cm-2).

7. Performance certification

Identity of the independent certification laboratory that confirmed the photovoltaic performance

☐ Yes *Identify the independent certification laboratory.*
☒ No This work does not report high power conversion efficiency values, but focuses on device physics.

A copy of any certificate(s)

☐ Yes *Certificate copies should be provided in the Supplementary information. Please state the supplementary item number.*
☒ No This work does not report high power conversion efficiency values, but focuses on device physics.

8. Statistics

Number of solar cells tested

☒ Yes Over 8 devices on more than 4 individual substrates were characterized for each active materials systems.
☐ No *Explain why this information is not reported/not relevant.*

Statistical analysis of the device performance

☒ Yes Data are provided in Table 1 and Table 2.
☐ No *Explain why this information is not reported/not relevant.*

9. Long-term stability analysis

Type of analysis, bias conditions and environmental conditions

☐ Yes *Provide a description of the type of analysis, bias conditions and environmental conditions (e.g. illumination type, temperature, atmosphere humidity, encapsulation method, preconditioning temperature, bias) for each long-term stability analysis carried out; see ref. 7 and 8 for details.*
☒ No The devices studied in this work are based on a standard device structure and commonly used active materials systems Stability studies on organic solar cells can be found in the literature.
